# Machine Learning to Predict Outcomes of Endovascular Intervention for Patients With PAD

**DOI:** 10.1001/jamanetworkopen.2024.2350

**Published:** 2024-03-14

**Authors:** Ben Li, Blair E. Warren, Naomi Eisenberg, Derek Beaton, Douglas S. Lee, Badr Aljabri, Raj Verma, Duminda N. Wijeysundera, Ori D. Rotstein, Charles de Mestral, Muhammad Mamdani, Graham Roche-Nagle, Mohammed Al-Omran

**Affiliations:** 1 Department of Surgery, University of Toronto, Toronto, Ontario, Canada; 2 Division of Vascular Surgery, St Michael’s Hospital, Unity Health Toronto, Toronto, Ontario, Canada; 3 Institute of Medical Science, University of Toronto, Toronto, Ontario, Canada; 4 Temerty Centre for Artificial Intelligence Research and Education in Medicine (T-CAIREM), University of Toronto, Toronto, Ontario, Canada; 5 Division of Vascular and Interventional Radiology, Department of Medical Imaging, University of Toronto, Toronto, Ontario, Canada; 6 Division of Vascular Surgery, Peter Munk Cardiac Centre, University Health Network, Toronto, Ontario, Canada; 7 Data Science & Advanced Analytics, Unity Health Toronto, University of Toronto, Toronto, Ontario, Canada; 8 Division of Cardiology, Peter Munk Cardiac Centre, University Health Network, Toronto, Ontario, Canada; 9 Institute of Health Policy, Management and Evaluation, University of Toronto, Toronto, Ontario, Canada; 10 ICES, University of Toronto, Toronto, Ontario, Canada; 11 Department of Surgery, King Saud University, Riyadh, Kingdom of Saudi Arabia; 12 School of Medicine, Royal College of Surgeons in Ireland, University of Medicine and Health Sciences, Dublin, Ireland; 13 Department of Anesthesia, St Michael’s Hospital, Unity Health Toronto, Toronto, Ontario, Canada; 14 Li Ka Shing Knowledge Institute, St Michael’s Hospital, Unity Health Toronto, Toronto, Ontario, Canada; 15 Division of General Surgery, St Michael’s Hospital, Unity Health Toronto, Toronto, Ontario, Canada; 16 Leslie Dan Faculty of Pharmacy, University of Toronto, Toronto, Ontario, Canada; 17 Department of Surgery, King Faisal Specialist Hospital and Research Center, Riyadh, Kingdom of Saudi Arabia

## Abstract

**Importance:**

Endovascular intervention for peripheral artery disease (PAD) carries nonnegligible perioperative risks; however, outcome prediction tools are limited.

**Objective:**

To develop machine learning (ML) algorithms that can predict outcomes following endovascular intervention for PAD.

**Design, Setting, and Participants:**

This prognostic study included patients who underwent endovascular intervention for PAD between January 1, 2004, and July 5, 2023, with 1 year of follow-up. Data were obtained from the Vascular Quality Initiative (VQI), a multicenter registry containing data from vascular surgeons and interventionalists at more than 1000 academic and community hospitals. From an initial cohort of 262 242 patients, 26 565 were excluded due to treatment for acute limb ischemia (n = 14 642) or aneurysmal disease (n = 3456), unreported symptom status (n = 4401) or procedure type (n = 2319), or concurrent bypass (n = 1747). Data were split into training (70%) and test (30%) sets.

**Exposures:**

A total of 112 predictive features (75 preoperative [demographic and clinical], 24 intraoperative [procedural], and 13 postoperative [in-hospital course and complications]) from the index hospitalization were identified.

**Main Outcomes and Measures:**

Using 10-fold cross-validation, 6 ML models were trained using preoperative features to predict 1-year major adverse limb event (MALE; composite of thrombectomy or thrombolysis, surgical reintervention, or major amputation) or death. The primary model evaluation metric was area under the receiver operating characteristic curve (AUROC). After selecting the best performing algorithm, additional models were built using intraoperative and postoperative data.

**Results:**

Overall, 235 677 patients who underwent endovascular intervention for PAD were included (mean [SD] age, 68.4 [11.1] years; 94 979 [40.3%] female) and 71 683 (30.4%) developed 1-year MALE or death. The best preoperative prediction model was extreme gradient boosting (XGBoost), achieving the following performance metrics: AUROC, 0.94 (95% CI, 0.93-0.95); accuracy, 0.86 (95% CI, 0.85-0.87); sensitivity, 0.87; specificity, 0.85; positive predictive value, 0.85; and negative predictive value, 0.87. In comparison, logistic regression had an AUROC of 0.67 (95% CI, 0.65-0.69). The XGBoost model maintained excellent performance at the intraoperative and postoperative stages, with AUROCs of 0.94 (95% CI, 0.93-0.95) and 0.98 (95% CI, 0.97-0.99), respectively.

**Conclusions and Relevance:**

In this prognostic study, ML models were developed that accurately predicted outcomes following endovascular intervention for PAD, which performed better than logistic regression. These algorithms have potential for important utility in guiding perioperative risk-mitigation strategies to prevent adverse outcomes following endovascular intervention for PAD.

## Key Points

**Question:**

What is the predictive performance of machine learning (ML) models for 1-year major adverse limb event (MALE) or death following endovascular intervention for peripheral artery disease (PAD)?

**Findings:**

In this prognostic study including 235 677 patients who underwent endovascular intervention for PAD, extreme gradient boosting achieved an area under the receiver operating characteristic curve of 0.94 for predicting 1-year MALE or death using preoperative features, outperforming logistic regression.

**Meaning:**

These findings suggest that ML models can accurately predict 1-year outcomes following endovascular intervention for PAD and have the potential to guide perioperative risk-mitigation strategies to improve outcomes.

## Teaser

This prognostic study assesses the ability of machine learning algorithms to predict outcomes following endovascular intervention for peripheral artery disease (PAD).

## Introduction

Peripheral artery disease (PAD) involves atherosclerosis of the lower extremity arteries, affecting more than 200 million individuals globally.^1,2,3,4^ Traditionally, advanced PAD was managed primarily with open surgery.^5^ In recent decades, endovascular intervention has become increasingly common as a minimally invasive alternative.^6^ Nevertheless, endovascular revascularization carries a high risk of complications, with major adverse limb events (MALE) or death occurring in more than half of the endovascular group in the BEST-CLI trial.^7^ Consequently, the Global Vascular Guidelines recommend careful risk assessment when considering patients for revascularization.^8^

Currently, there are no widely used tools to predict complications following endovascular intervention for PAD. In the research setting, prediction models are limited to trauma patients,^9^ Japanese or Spanish cohorts,^10,11^ and open revascularization.^12,13^ Furthermore, tools such as the National Surgical Quality Improvement Program (NSQIP) online surgical risk calculator^14^ rely on modeling techniques that require manual input of clinical variables, which deters routine use.^15^ Therefore, there is a need to develop better risk prediction tools for patients being considered for endovascular revascularization.

Machine learning (ML) is a rapidly evolving technology that empowers computers to learn from data and make accurate predictions.^16^ Bonde and colleagues^17^ previously used NSQIP data to develop ML algorithms that predict perioperative complications in a pooled data set of more than 2900 unique procedures. Given the heterogeneity of this cohort, better predictive performance may be achieved by building ML algorithms specific to patients undergoing endovascular intervention for PAD using the Society for Vascular Surgery (SVS) Vascular Quality Initiative (VQI) database, a dedicated vascular registry containing procedure-specific variables. In this study, we used VQI data to develop ML algorithms that predict 1-year MALE or death following endovascular intervention for PAD.

## Methods

### Study Approval and Design

The SVS Patient Safety Organization (PSO) Research Advisory Council approved this project and provided the deidentified data set. Patient consent was not required as the data originated from an anonymized registry. This was an ML-based prognostic study reported according to the Transparent Reporting of a Multivariable Prediction Model for Individual Prognosis or Diagnosis (TRIPOD) statement.^18^

### Dataset

The VQI database is a clinical registry maintained by the SVS PSO with the goal of improving vascular care.^19^ Vascular surgeons, interventionalists, and other specialists across more than 1000 academic and community hospitals in the United States, Canada, and Singapore prospectively contribute demographic, clinical, and outcomes data on consecutive eligible vascular patients including information from their index procedure up to approximately 1 year of follow-up.^19^ Routine audits comparing submitted data with hospital claims are performed to ensure data accuracy.^20^

### Patient Cohort

All patients who underwent endovascular intervention for lower extremity PAD (suprainguinal or infrainguinal angioplasty, stent, and/or atherectomy) from January 1, 2004, to July 5, 2023, in the VQI database were included. Patients treated for lower extremity aneurysmal disease, acute limb ischemia, trauma, dissection, or malignant neoplasm, and those with unreported symptom status or procedure type, or undergoing concurrent surgical bypass were excluded.

### Features

Predictor variables (features) used in the ML models were divided into preoperative, intraoperative, and postoperative stages. Given the advantage of ML in handling many input features, all available variables in the VQI database were used to maximize predictive performance. Given the goal of automated extraction of features rather than manual input of clinical variables to generate risk predictions, feature selection was not performed to maximize predictive performance. Preoperative features (n = 75) included demographic characteristics, comorbidities, previous procedures, functional status, investigations, medications, anatomy, and other preprocedural characteristics. Given the relatively long cohort recruitment period, the year of intervention was included as an input feature to account for its potential association with outcomes. Intraoperative features (n = 24) included access artery, adjunctive procedures, use of procedural anticoagulation and/or protamine, contrast volume, fluoroscopy time, and technical success (≤30% residual stenosis). Postoperative features (n = 13) included in-hospital characteristics: cardiac, pulmonary, kidney, access site, and contrast complications; length of stay; discharge medications; and nonhome discharge. A complete list of features and their definitions can be found in eTables 1 to 3 in Supplement 1. Given potential for variability in definitions among clinicians, we recommend adhering to established clinical guidelines with regards to clinical definitions.^8,21^

### Outcomes

The primary outcome was 1-year postprocedural major adverse limb event (MALE; composite of thrombectomy or thrombolysis, surgical reintervention, or major amputation) or death. Thrombectomy or thrombolysis was defined as surgical or catheter-based thrombectomy or thrombolysis of the treated segment. Surgical reintervention was defined as an open reintervention on the treated segment including interposition graft or bypass. Major amputation was defined as a below-knee or more proximal amputation of the ipsilateral leg. Death was defined as all-cause mortality. The composite outcome of MALE or death was chosen because it is commonly reported as the primary outcome in key clinical trials, including BEST-CLI.^7^ Secondary outcomes were individual components of the primary outcome. These definitions were based on the VQI data dictionary.^19^

### Model Development

We trained 6 different ML models to predict primary and secondary outcomes: extreme gradient boosting (XGBoost), random forest, naive Bayes classifier, radial basis function (RBF) support vector machine (SVM), multilayer perceptron (MLP) artificial neural network (ANN), and logistic regression. The data were randomly divided into training (70%) and testing (30%) sets. To determine the optimal model hyperparameters, 10-fold cross-validation and grid search were applied to training data.^22,23^ Initial analysis demonstrated that the primary outcome occurred in 71 683 of 235 677 patients (30.4%) in our cohort. To improve class balance, random oversample examples (ROSE) were applied to training data.^24^ The models were then evaluated on test set data and ranked based on the primary discriminatory metric of area under the receiver operating characteristic curve (AUROC). The models were compared at the preoperative stage because predictions at this time offer the most potential to mitigate adverse events, such as deciding whether to proceed with intervention.^25^ After identifying the best performing ML model at the preoperative stage, we further trained the algorithm using intraoperative and postoperative data, as detailed in eFigure 1 in Supplement 1. This approach allows clinicians to gain insights into a patient’s risk at different stages of their perioperative course, thereby guiding decision-making before, during, and after intervention.

### Statistical Analysis

Preoperative, intraoperative, and postoperative features were summarized as means and SDs for continuous variables and numbers and percentages for categorical variables. Differences between groups were assessed using independent *t* tests (continuous variables) and χ^2^ tests (categorical variables). The primary model evaluation metric was AUROC. Secondary performance metrics were accuracy, sensitivity, specificity, positive predictive value (PPV), and negative predictive value (NPV). To assess model robustness, we plotted calibration curves and calculated Brier scores.^26^ In the final model, feature importance was determined by ranking the top 10 predictors based on variable importance scores (gain).^27^ Feature importance was determined for the overall cohort, patients with chronic limb-threatening ischemia (CLTI), and asymptomatic and claudication groups. To assess model bias, we evaluated predictive performance across subgroups based on age, sex, race, ethnicity, rurality, median Area Deprivation Index (ADI) percentile, symptom status, procedure type, primary artery treated, prior intervention for PAD, procedure setting, and urgency.

 Race and ethnicity were determined through participant self-report. Participants could select from American Indian or Alaskan Native, Asian, Black, Native Hawaiian or Other Pacific Islander, White, more than 1 race, or unknown or other for race and from Hispanic or non-Hispanic for ethnicity. These racial and ethnic groups were determined based on the US Census Bureau. Race and ethnicity were included because of their previously demonstrated associations with PAD outcomes.^28^

To achieve a minimum AUROC of 0.7 in a cohort with an outcome rate of approximately 30% and 75 pre-operative features, a minimum sample size of 6178 patients with 1854 events is required.^29^ Our cohort of 235 677 patients with 71 683 primary events satisfied this requirement. For variables of interest, missing data was less than 5%. Therefore, we used a complete-case analysis approach, considering only nonmissing covariates for each patient. This is a valid approach for datasets with minimal missing data (<5%).^30,31^ Patients lost to follow-up were censored. Specifically, we applied a general-purpose approach to account for right-censored outcomes using inverse probability of censoring weighting as described by Vock et al.^32^ Statistical significance was set at 2-tailed *P* < .05. All analyses were conducted using R version 4.3.1 (R Project for Statistical Computing).

## Results

### Patients, Events, and Follow-Up

From an initial cohort of 262 242 patients, a total of 26 565 were excluded for the following reasons: treatment for acute limb ischemia (n = 14 642), treatment for aneurysmal disease (n = 3456), unreported symptom status (n = 4401), unreported procedure type (n = 2319), and concurrent bypass for PAD (n = 1747). The final analysis included 235 677 patients (mean [SD] age, 68.4 [11.1] years; 94 979 [40.3%] female), of whom 71 683 (30.4%) developed 1-year MALE or death. The secondary outcomes occurred in the following distribution: thrombectomy or thrombolysis, 25 303 patients (10.7%); surgical reintervention, 7326 patients (3.1%); major amputation, 13 938 patients (5.9%); and death, 32 892 patients (14.0%). Mean (SD) follow-up was 16.1 (5.7) months.

### Preoperative Characteristics

Compared with patients without a primary outcome, those who developed 1-year MALE or death were older and more likely to be female, Black, and Hispanic; more likely to receive Medicare; and more likely to have a higher median ADI. They were also more likely to have hypertension, diabetes, coronary artery disease, dysrhythmia, congestive heart failure, prior stroke, chronic obstructive pulmonary disease, and end-stage kidney disease requiring dialysis, yet less likely to receive acetylsalicylic acid (ASA), statin, and angiotensin converting enzyme inhibitor (ACE-I) or angiotensin II receptor blocker (ARB) preoperatively. Anatomically, patients with an outcome had a greater median number of arteries treated, with a higher proportion being tibial; longer occlusion or treatment lengths; and lesions that were Trans-Atlantic Society Consensus (TASC) grade C or D. Patients with MALE or death at 1 year were more likely to have CLTI and require urgent or emergent intervention (eTable 5 in Supplement 1).

### Intraoperative Characteristics

Patients with 1-year MALE or death were more likely to undergo adjunctive procedures including thrombolysis, suction thrombectomy, and femoral endarterectomy. Patients with an event had a higher median fluoroscopy time and a lower total contrast volume. Technical success rates were lower in patients who reached the primary end point (eTable 6 in Supplement 1).

### Postoperative Characteristics

A greater proportion of patients with 1-year MALE or death had in-hospital cardiac, pulmonary, kidney, and access site complications, yet they were less likely to be discharged receiving ASA, P2Y12 antagonists, statins, and ACE-I/ARB. The median length of stay was higher for patients with an event, and they were more likely to undergo nonhome discharge (eTable 7 in Supplement 1).

### Model Performance

Of the 6 ML models assessed at the preoperative stage using test set data, XGBoost had the best performance in predicting 1-year MALE or death (AUROC, 0.94 [95% CI, 0.93-0.95]). In comparison, the other models had the following AUROCs: random forest, 0.92 (95% CI, 0.91-0.93); naive Bayes, 0.84 (95% CI, 0.83-0.85); MLP ANN, 0.80 (95% CI, 0.78-0.82); RBF SVM, 0.78 (95% CI, 0.78-0.80); and logistic regression, 0.67 (95% CI, 0.65-0.69). Model performance results are summarized in the Table. The XGBoost had an accuracy of 0.86 (95% CI, 0.85-0.87); sensitivity, 0.87; specificity, 0.85; PPV, 0.85; and NPV, 0.87. XGBoost hyperparameters are detailed in eTable 4 in Supplement 1.

**Table.** Model Performance on Test Set Data for Predicting 1-Year Major Adverse Limb Event or Death Following Endovascular Intervention for Peripheral Artery Disease Using Preoperative Features

Model	AUROC (95% CI)	Accuracy (95% CI)	Sensitivity	Specificity	PPV	NPV
XGBoost	0.94 (0.93-0.95)	0.86 (0.85-0.87)	0.87	0.85	0.85	0.87
Random forest	0.92 (0.91-0.93)	0.84 (0.83-0.85)	0.87	0.81	0.80	0.88
Naive Bayes	0.84 (0.83-0.85)	0.84 (0.83-0.85)	0.83	0.85	0.86	0.81
MLP ANN	0.80 (0.78-0.82)	0.75(0.73-0.76)	0.77	0.72	0.71	0.78
RBF SVM	0.78 (0.77-0.80)	0.71 (0.70-0.73)	0.71	0.71	0.72	0.70
Logistic regression	0.67 (0.65-0.69)	0.60 (0.58-0.61)	0.57	0.71	0.67	0.52

Abbreviations: AUROC, area under the receiver operating characteristic curve; MLP ANN, multilayer perceptron artificial neural network; NPV, negative predictive value; PPV, positive predictive value; RBF SVM, radial basis function support vector machine; XGBoost, extreme gradient boosting.

**Figure 1.**
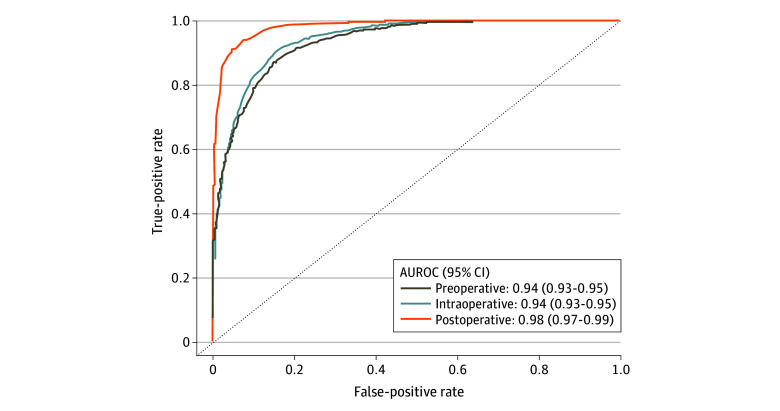
Receiver Operating Characteristic Curve for Predicting 1-Year Major Adverse Limb Event or Death Following Endovascular Intervention for Peripheral Artery Disease Using Extreme Gradient Boosting Models at the Preoperative, Intraoperative, and Postoperative Stages AUROC indicates area under the receiver operating characteristic curve.

**Figure 2.**
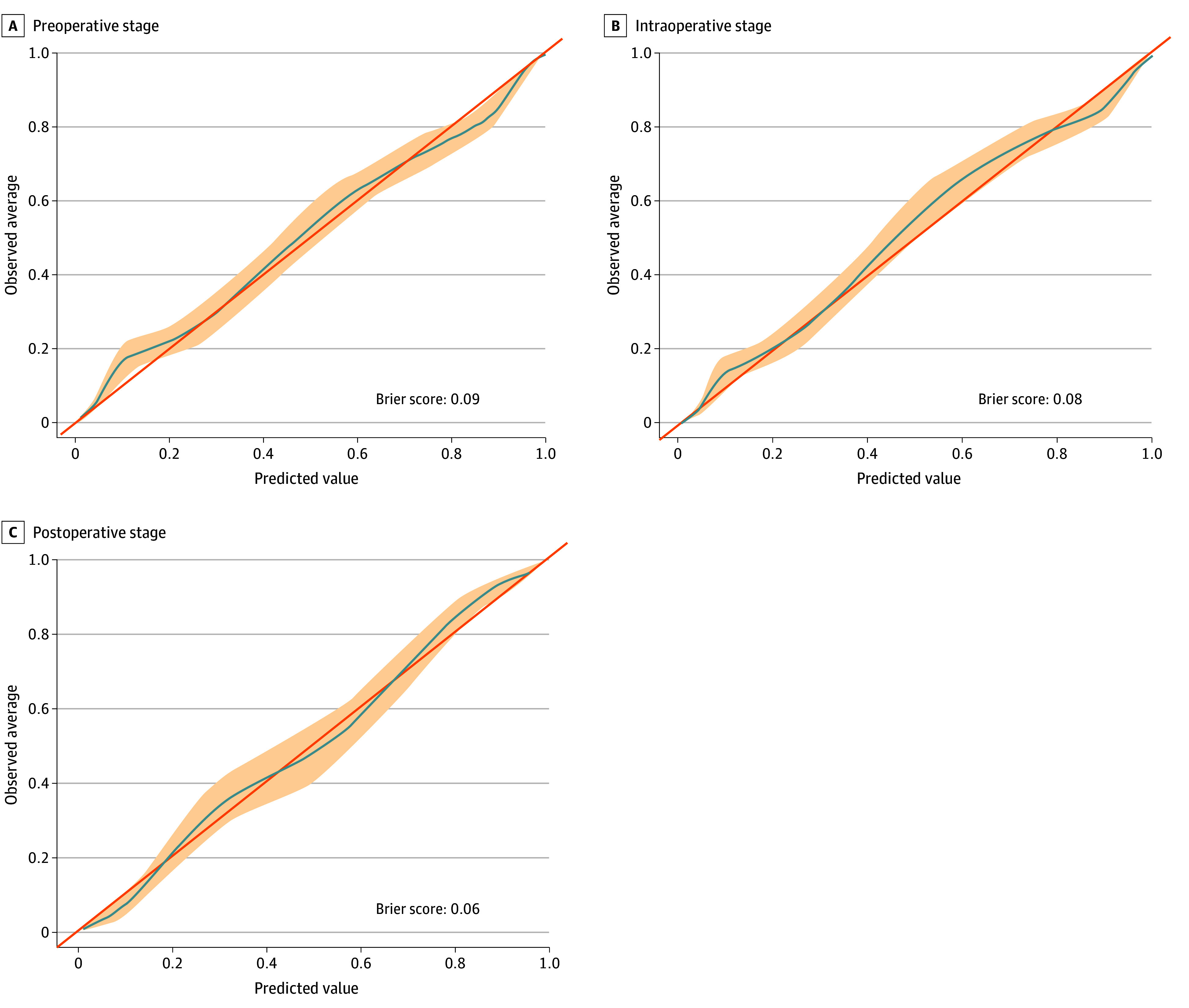
Calibration Plots With Brier Scores for Predicting 1-Year Major Adverse Limb Event or Death Following Endovascular Intervention for Peripheral Artery Disease Using Extreme Gradient Boosting Models at the Preoperative, Intraoperative, and Postoperative Stages

We further trained the XGBoost model using intraoperative and postoperative data. The addition of intraoperative features did not significantly change performance, with the AUROC remaining at 0.94 (95% CI, 0.93-0.95). Adding postoperative features improved performance to an AUROC of 0.98 (95% CI, 0.97-0.99). The ROC curves are presented in Figure 1. There was good agreement between predicted and observed event probabilities, as demonstrated by the calibration plots in Figure 2, with Brier scores of 0.09 (preoperative), 0.08 (intraoperative), and 0.06 (postoperative). XGBoost predicted individual components of the primary outcome with the following AUROC ranges: 0.91 to 0.94 (preoperative), 0.93 to 0.95 (intraoperative), and 0.95 to 0.98 (postoperative) (eTable 8 in Supplement 1).

**Figure 3.**
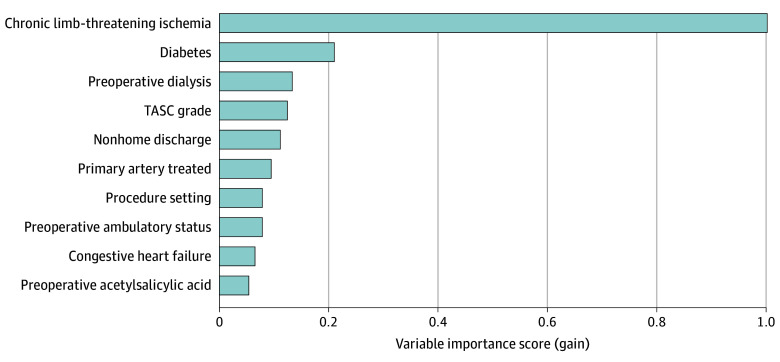
Variable Importance Scores for the Top 10 Predictors of 1-Year Major Adverse Limb Event or Death Following Endovascular Intervention for Peripheral Artery Disease in the Extreme Gradient Boosting Model at the Postoperative Stage TASC indicates Trans-Atlantic Society Consensus.

The top 10 predictors of 1-year MALE or death in the final XGBoost model included 9 preoperative features (CLTI, diabetes, preoperative dialysis, TASC grade, primary artery treated, procedure setting, preoperative ambulatory status, congestive heart failure, and preoperative receipt of ASA) and 1 postoperative feature (nonhome discharge) (Figure 3). On subgroup analysis based on symptom status, 8 of 10 of the most important predictive features were the same for patients with CLTI and those who were asymptomatic or had claudication (eFigure 2 in Supplement 1).

### Subgroup Analysis

Model performance remained robust across demographic and clinical subpopulations, with AUROCs ranging from 0.93 to 0.94. There were no significant differences between majority and minority groups (eFigures 3-14 in Supplement 1).

## Discussion

We leveraged data from a large clinical registry consisting of 235 677 patients who underwent endovascular intervention for PAD to develop ML models that accurately predicted 1-year postprocedural MALE or death with AUROCs of greater than 0.90. We evaluated 6 ML models, and XGBoost achieved the best performance. The XGBoost algorithm demonstrated excellent discrimination and calibration across the preoperative, intraoperative, and postoperative stages. Furthermore, predictive performance remained robust across demographic and clinical subpopulations. These models have the potential to support clinical decision-making throughout a patient’s perioperative course by facilitating individualized risk assessment.

Bertges and colleagues^13^ developed the VQI Cardiac Risk Index to predict in-hospital myocardial infarction in patients undergoing lower extremity bypass, carotid endarterectomy, and aortic aneurysm repair. Using logistic regression, their model achieved an AUROC of 0.75.^13^ Importantly, endovascular intervention for PAD was not included.^13^ More recently, Simons and colleagues^33^ applied Cox regression to predict long-term mortality in patients undergoing infrainguinal bypass or endovascular intervention for CLTI using VQI data, achieving AUROCs between 0.71 and 0.76. Applying ML techniques to a more up-to-date cohort exclusively composed of patients undergoing endovascular intervention for PAD, we achieved better performance with AUROCs greater than 0.90 for predicting 1-year MALE or death, a more clinically relevant limb-related outcome.

Several factors contribute to the interpretation of our findings. First, despite multiple societal guideline recommendations for antiplatelet and statin therapy in patients with PAD,^8,21,34,35^ our study showed that individuals who developed complications were less likely to receive antiplatelets or statins preoperatively and at discharge. Therefore, there are opportunities to improve PAD care by understanding patients’ perioperative risk and optimizing them before and after revascularization. There may be several factors contributing to suboptimal rates of best medical therapy, including lack of access to care, drug insurance coverage, or clinician time and education, among other factors.^36,37^ There may be opportunities to improve rates of best medical therapy along with physical activity through patient education in the clinic, PAD awareness campaigns, and further integration of PAD education into medical school curricula.^38,39^ Second, we found that preoperative factors were the most important predictors of 1-year MALE or death, while intraoperative features did not significantly improve model performance. This suggests that the ability to accurately predict long-term outcomes can be established early in the perioperative course. The limited impact of intraoperative factors on long-term risk may be attributed to the standardization of endovascular principles over time.^40^ Third, our ML models performed better than existing tools for several reasons. Compared with logistic regression, advanced ML techniques can better model nonlinear, complex associations between inputs and outputs.^41,42^ This is important in health care data, as patient outcomes can be affected by many demographic and clinical factors.^43^ Our top-performing algorithm was XGBoost, which has advantages, including relatively fewer issues with overfitting and faster computing while maintaining precision.^44,45,46^ Fourth, our model performance remained robust across demographic and clinical subpopulations. Importantly, our analysis demonstrated associations between various demographic factors including age, sex, race, ethnicity, insurance status, ADI, and procedure setting and postoperative outcomes. Given prior literature demonstrating the disproportionate impact of PAD on disadvantaged and minority populations, it is critical for future work to carefully consider aspects of diversity, equity, and inclusion in the management of patients with PAD.^28,47^ Particularly, an increasing focus on addressing systemic and structural inequity related to social determinants of health, including economic stability, education, and access to health care, will be critical to improving PAD outcomes.^48^

**Figure 4.**
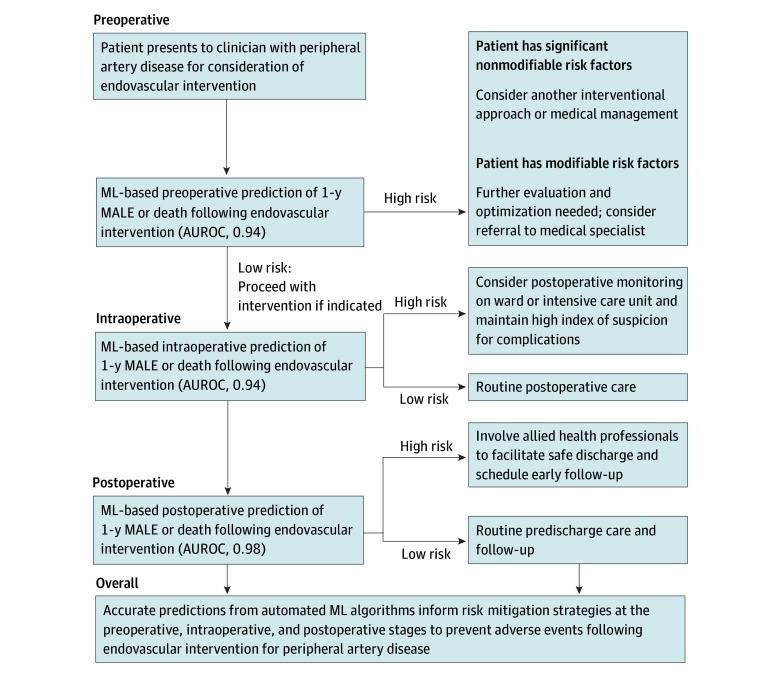
Clinical Workflow for the Use of Machine Learning (ML) Algorithms to Guide Clinical Decision-Making at the Preoperative, Intraoperative, and Postoperative Stages for Patients Being Considered for Endovascular Intervention for Peripheral Artery Disease High risk defined as a model prediction positive for 1-year major adverse limb event (MALE) or death. Low risk defined as a model prediction negative for 1-year MALE or death. AUROC indicates area under the receiver operating characteristic curve.

Our ML models can be applied in several ways. Preoperatively, a patient predicted to be at high risk for adverse events should be further evaluated.^49^ For those with nonmodifiable risks, alternative management options, such as medical therapy alone or other interventional approaches, should be considered.^40,50^ Patients with modifiable risks may benefit from further evaluation and optimization with appropriate referrals to medical specialists including cardiologists and internists.^51,52^ Postoperatively, high-risk patients should be closely monitored to provide timely intervention if complications arise.^53^ Patients at high risk of long-term complications should receive early support from allied health professionals to optimize safe discharge planning with consideration of early follow-up.^54^ Given the important role of social determinants of health in postoperative outcomes, it is critical to effectively follow-up with high-risk patients postoperatively and implement strategies for proactive and multidisciplinary care, including physiotherapy, interdisciplinary treatment facilities, and regular follow-up phone calls to mitigate adverse events.^55,56^ Figure 4 illustrates a proposed clinical workflow for our ML tool to support clinical decision-making at the preoperative, intraoperative, and postoperative stages.

The programming code used to develop our models is publicly available on GitHub. At a system-wide level, our models can be implemented by the more than 1000 VQI participating centers.^19^ The number of VQI centers has grown considerably from 400 in 2019 to more than 1000 in 2023.^19,57^ Therefore, our models have broad and growing utility. A key advantage of our ML models is their ability to provide automated risk predictions, thereby enhancing feasibility in busy clinical settings compared with traditional risk predictors that often require manual input of variables.^14^ Specifically, our ML algorithms can autonomously extract a patient’s VQI data to generate risk predictions. Ongoing work is being performed to link electronic health records to registry data such as VQI, which would further increase the automation capabilities and potential clinical utility of our model.^58^ To facilitate successful implementation of our ML tool, we recommend establishing data analytics teams at the institutional level.^59^

### Limitations

Our study has several limitations. First, our models were developed with VQI data, a voluntary registry primarily comprising North American data. Future studies are needed to assess whether performance can be generalized beyond VQI sites. Second, our models are limited to patients undergoing endovascular intervention for PAD. We previously reported a NSQIP-based ML algorithm for predicting 30-day outcomes following open aortoiliac revascularization,^60^ and development of ML models for other PAD interventions is under way by our group. Third, complete-case analysis was performed due to a small amount of missing data for variables of interest (<5%). Given that any missing data may introduce bias, future work aimed at reducing the missingness of data may further augment the performance of predictive models. Furthermore, prospective validation of our model on real-world data, where data missingness may be greater, is important to assess how missing data may affect model performance.

## Conclusions

We used a large, vascular-specific clinical registry to develop robust ML models that predict 1-year MALE/death following endovascular intervention for PAD with excellent performance (AUROCs >0.90). Our models can be applied across the preoperative, intraoperative, and postoperative stages to guide clinical decision-making regarding risk-mitigation strategies. Notably, our models remained robust across demographic and clinical subpopulations and outperformed existing prediction tools and logistic regression and therefore, have potential for important utility in the care of patients with PAD. Prospective clinical validation of our ML algorithms is warranted to assess practical utility and integration into clinical workflows.

## Notes

Supplement 1. **eTable 1.** Preoperative Features for Machine Learning Models **eTable 2.** Intraoperative Features for Machine Learning Models **eTable 3.** Postoperative Features for Machine Learning Models **eTable 4.** Selection of Extreme Gradient Boosting (XGBoost) Model Hyperparameters Using Grid Search and Cross-Validation **eTable 5.** Preoperative Demographic and Clinical Characteristics of Patients Undergoing Endovascular Intervention for Peripheral Artery Disease With and Without Major Adverse Limb Event or Death at 1 Year **eTable 6.** Intraoperative Characteristics of Patients Undergoing Endovascular Intervention for Peripheral Artery Disease With and Without Major Adverse Limb Event or Death at 1 Year **eTable 7.** Postoperative In-Hospital Characteristics and Complications of Patients Undergoing Endovascular Intervention for Peripheral Artery Disease With and Without Major Adverse Limb Event or Death at 1 Year **eTable 8.** XGBoost Performance on Test Set Data for Predicting 1-Year Primary and Secondary Outcomes Following Endovascular Intervention for Peripheral Artery Disease at the Preoperative, Intraoperative, and Postoperative Stages **eFigure 1.** Variables Considered at the Preoperative, Intraoperative, and Postoperative Stages for Model Development **eFigure 2.** Variable Importance Scores (Gain) for the Top 10 Predictors of 1-Year Major Adverse Limb Event or Death Following Endovascular Intervention for Peripheral Artery Disease in the Extreme Gradient Boosting (XGBoost) Model at the Postoperative Stage With Subgroup Analysis Based on Symptom Status **eFigure 3.** Receiver Operating Characteristic Curve for Predicting 1-Year Major Adverse Limb Event or Death Following Endovascular Intervention for Peripheral Artery Disease Using Extreme Gradient Boosting (XGBoost) Model at the Preoperative Stage With Subgroup Analysis Based on Age **eFigure 4.** Receiver Operating Characteristic Curve for Predicting 1-Year Major Adverse Limb Event or Death Following Endovascular Intervention for Peripheral Artery Disease Using Extreme Gradient Boosting (XGBoost) Model at the Preoperative Stage With Subgroup Analysis Based on Sex **eFigure 5.** Receiver Operating Characteristic Curve for Predicting 1-Year Major Adverse Limb Event or Death Following Endovascular Intervention for Peripheral Artery Disease Using Extreme Gradient Boosting (XGBoost) Model at the Preoperative Stage With Subgroup Analysis Based on Race **eFigure 6.** Receiver Operating Characteristic Curve for Predicting 1-Year Major Adverse Limb Event or Death Following Endovascular Intervention for Peripheral Artery Disease Using Extreme Gradient Boosting (XGBoost) Model at the Preoperative Stage With Subgroup Analysis Based on Ethnicity **eFigure 7.** Receiver Operating Characteristic Curve for Predicting 1-Year Major Adverse Limb Event or Death Following Endovascular Intervention for Peripheral Artery Disease Using Extreme Gradient Boosting (XGBoost) Model at the Preoperative Stage With Subgroup Analysis Based on Rurality of Residence **eFigure 8.** Receiver Operating Characteristic Curve for Predicting 1-Year Major Adverse Limb Event or Death Following Endovascular Intervention for Peripheral Artery Disease Using Extreme Gradient Boosting (XGBoost) Model at the Preoperative Stage With Subgroup Analysis Based on Median Area Deprivation Index (ADI) Percentile **eFigure 9.** Receiver Operating Characteristic Curve for Predicting 1-Year Major Adverse Limb Event or Death Following Endovascular Intervention for Peripheral Artery Disease Using Extreme Gradient Boosting (XGBoost) Model at the Preoperative Stage With Subgroup Analysis Based on Symptom Status **eFigure 10.** Receiver Operating Characteristic Curve for Predicting 1-Year Major Adverse Limb Event or Death Following Endovascular Intervention for Peripheral Artery Disease Using Extreme Gradient Boosting (XGBoost) Model at the Preoperative Stage With Subgroup Analysis Based on Primary Procedure Type **eFigure 11.** Receiver Operating Characteristic Curve for Predicting 1-Year Major Adverse Limb Event or Death Following Endovascular Intervention for Peripheral Artery Disease Using Extreme Gradient Boosting (XGBoost) Model at the Preoperative Stage With Subgroup Analysis Based on Location of Primary Artery Treated **eFigure 12.** Receiver Operating Characteristic Curve for Predicting 1-Year Major Adverse Limb Event or Death Following Endovascular Intervention for Peripheral Artery Disease (PAD) Using Extreme Gradient Boosting (XGBoost) Model at the Preoperative Stage With Subgroup Analysis Based on Presence of Prior Open or Endovascular Intervention for PAD **eFigure 13.** Receiver Operating Characteristic Curve for Predicting 1-Year Major Adverse Limb Event or Death Following Endovascular Intervention for Peripheral Artery Disease (PAD) Using Extreme Gradient Boosting (XGBoost) Model at the Preoperative Stage With Subgroup Analysis Based on Procedure Setting **eFigure 14.** Receiver Operating Characteristic Curve for Predicting 1-Year Major Adverse Limb Event or Death Following Endovascular Intervention for Peripheral Artery Disease (PAD) Using Extreme Gradient Boosting (XGBoost) Model at the Preoperative Stage With Subgroup Analysis Based on Urgency

Supplement 2.
Data Sharing Statement
