# Supplementary material for: Machine Learning to Predict Outcomes of Endovascular Intervention for Patients With PAD
Source: JAMA Netw Open. 2024 Mar 14;7(3):e242350. doi: 10.1001/jamanetworkopen.2024.2350 (PMC10940965; doi:10.1001/jamanetworkopen.2024.2350)
Supplement: Supplement 1. — eTable 1. Preoperative Features for Machine Learning Models eTable 2. Intraoperative Features for Machine Learning Models eTable 3. Postoperative Features for Machine Learning Models eTable 4. Selection of Extreme Gradient Boosting (XGBoost) Model Hyperparameters Using Grid Search and Cross-Validation eTable 5. Preoperative Demographic and Clinical Characteristics of Patients Undergoing Endovascular Intervention for Peripheral Artery Disease With and Without Major Adverse Limb Event or Death at 1 Year eTable 6. Intraoperative Characteristics of Patients Undergoing Endovascular Intervention for Peripheral Artery Disease With and Without Major Adverse Limb Event or Death at 1 Year eTable 7. Postoperative In-Hospital Characteristics and Complications of Patients Undergoing Endovascular Intervention for Peripheral Artery Disease With and Without Major Adverse Limb Event or Death at 1 Year eTable 8. XGBoost Performance on Test Set Data for Predicting 1-Year Primary and Secondary Outcomes Following Endovascular Intervention for Peripheral Artery Disease at the Preoperative, Intraoperative, and Postoperative Stages eFigure 1. Variables Considered at the Preoperative, Intraoperative, and Postoperative Stages for Model Development eFigure 2. Variable Importance Scores (Gain) for the Top 10 Predictors of 1-Year Major Adverse Limb Event or Death Following Endovascular Intervention for Peripheral Artery Disease in the Extreme Gradient Boosting (XGBoost) Model at the Postoperative Stage With Subgroup Analysis Based on Symptom Status eFigure 3. Receiver Operating Characteristic Curve for Predicting 1-Year Major Adverse Limb Event or Death Following Endovascular Intervention for Peripheral Artery Disease Using Extreme Gradient Boosting (XGBoost) Model at the Preoperative Stage With Subgroup Analysis Based on Age eFigure 4. Receiver Operating Characteristic Curve for Predicting 1-Year Major Adverse Limb Event or Death Following Endovascular Intervention for Peripheral Artery Diseas [file jamanetwopen-e242350-s001.pdf]

Supplemental Online Content

Li B, Warren BE, Eisenberg N, et al. Machine learning to predict outcomes of endovascular intervention for patients with PAD. *JAMA Netw Open.* 2024;7(3):e242350. doi:10.1001/jamanetworkopen.2024.2350

eTable 1. Preoperative Features for Machine Learning Models

eTable 2. Intraoperative Features for Machine Learning Models

eTable 3. Postoperative Features for Machine Learning Models

eTable 4. Selection of Extreme Gradient Boosting (XGBoost) Model Hyperparameters Using Grid Search and Cross-Validation

eTable 5. Preoperative Demographic and Clinical Characteristics of Patients Undergoing Endovascular Intervention for Peripheral Artery Disease With and Without Major Adverse Limb Event or Death at 1 Year

eTable 6. Intraoperative Characteristics of Patients Undergoing Endovascular Intervention for Peripheral Artery Disease With and Without Major Adverse Limb Event or Death at 1 Year

eTable 7. Postoperative In-Hospital Characteristics and Complications of Patients Undergoing Endovascular Intervention for Peripheral Artery Disease With and Without Major Adverse Limb Event or Death at 1 Year

eTable 8. XGBoost Performance on Test Set Data for Predicting 1-Year Primary and Secondary Outcomes Following Endovascular Intervention for Peripheral Artery Disease at the Preoperative, Intraoperative, and Postoperative Stages

eFigure 1. Variables Considered at the Preoperative, Intraoperative, and Postoperative Stages for Model Development

eFigure 2. Variable Importance Scores (Gain) for the Top 10 Predictors of 1-Year Major Adverse Limb Event or Death Following Endovascular Intervention for Peripheral Artery Disease in the Extreme Gradient Boosting (XGBoost) Model at the Postoperative Stage With Subgroup Analysis Based on Symptom Status

eFigure 3. Receiver Operating Characteristic Curve for Predicting 1-Year Major Adverse Limb Event or Death Following Endovascular Intervention for Peripheral Artery Disease Using Extreme Gradient Boosting (XGBoost) Model at the Preoperative Stage With Subgroup Analysis Based on Age

eFigure 4. Receiver Operating Characteristic Curve for Predicting 1-Year Major Adverse Limb Event or Death Following Endovascular Intervention for Peripheral Artery Disease Using Extreme Gradient Boosting (XGBoost) Model at the Preoperative Stage With Subgroup Analysis Based on Sex

eFigure 5. Receiver Operating Characteristic Curve for Predicting 1-Year Major Adverse Limb Event or Death Following Endovascular Intervention for Peripheral Artery Disease Using

© 2024 Li B et al. *JAMA Network Open.*

Extreme Gradient Boosting (XGBoost) Model at the Preoperative Stage With Subgroup Analysis Based on Race

eFigure 6. Receiver Operating Characteristic Curve for Predicting 1-Year Major Adverse Limb Event or Death Following Endovascular Intervention for Peripheral Artery Disease Using Extreme Gradient Boosting (XGBoost) Model at the Preoperative Stage With Subgroup Analysis Based on Ethnicity

eFigure 7. Receiver Operating Characteristic Curve for Predicting 1-Year Major Adverse Limb Event or Death Following Endovascular Intervention for Peripheral Artery Disease Using Extreme Gradient Boosting (XGBoost) Model at the Preoperative Stage With Subgroup Analysis Based on Rurality of Residence

eFigure 8. Receiver Operating Characteristic Curve for Predicting 1-Year Major Adverse Limb Event or Death Following Endovascular Intervention for Peripheral Artery Disease Using Extreme Gradient Boosting (XGBoost) Model at the Preoperative Stage With Subgroup Analysis Based on Median Area Deprivation Index (ADI) Percentile

eFigure 9. Receiver Operating Characteristic Curve for Predicting 1-Year Major Adverse Limb Event or Death Following Endovascular Intervention for Peripheral Artery Disease Using Extreme Gradient Boosting (XGBoost) Model at the Preoperative Stage With Subgroup Analysis Based on Symptom Status

eFigure 10. Receiver Operating Characteristic Curve for Predicting 1-Year Major Adverse Limb Event or Death Following Endovascular Intervention for Peripheral Artery Disease Using Extreme Gradient Boosting (XGBoost) Model at the Preoperative Stage With Subgroup Analysis Based on Primary Procedure Type

eFigure 11. Receiver Operating Characteristic Curve for Predicting 1-Year Major Adverse Limb Event or Death Following Endovascular Intervention for Peripheral Artery Disease Using Extreme Gradient Boosting (XGBoost) Model at the Preoperative Stage With Subgroup Analysis Based on Location of Primary Artery Treated

eFigure 12. Receiver Operating Characteristic Curve for Predicting 1-Year Major Adverse Limb Event or Death Following Endovascular Intervention for Peripheral Artery Disease (PAD) Using Extreme Gradient Boosting (XGBoost) Model at the Preoperative Stage With Subgroup Analysis Based on Presence of Prior Open or Endovascular Intervention for PAD

eFigure 13. Receiver Operating Characteristic Curve for Predicting 1-Year Major Adverse Limb Event or Death Following Endovascular Intervention for Peripheral Artery Disease (PAD) Using Extreme Gradient Boosting (XGBoost) Model at the Preoperative Stage With Subgroup Analysis Based on Procedure Setting

eFigure 14. Receiver Operating Characteristic Curve for Predicting 1-Year Major Adverse Limb Event or Death Following Endovascular Intervention for Peripheral Artery Disease (PAD) Using Extreme Gradient Boosting (XGBoost) Model at the Preoperative Stage With Subgroup Analysis Based on Urgency

This supplemental material has been provided by the authors to give readers additional information about their work.

eTable 1. Preoperative Features for Machine Learning Models

Features (n = 75)	Definition based on Vascular Quality Initiative Data Dictionary
**Logistics**	
Region	Region of procedure (deidentified)
Center	Center of procedure (deidentified)
Physician	Physician who performed procedure (deidentified)
Intervention year	Year of intervention
Intervention month	Month of intervention
Intervention weekday	Day of week that intervention was performed
**Demographics**	
Age	Age in years
Sex	Male or female
Body mass index	Weight in kg / height in m^2
Race	Patient-reported race including White, Black, Asian, American Indian or Alaskan Native, Native Hawaiian or other Pacific Islander, more than 1 race, or unknown/other
Ethnicity	Hispanic or non-Hispanic
Primary insurer	Primary method of health insurance including Medicare, Medicaid, Commercial, Medicare Advantage, Military or Veterans Affairs, Non-US Insurance, self-pay (uninsured), or unknown/other
Rural residence	Defined based on the patient's primary rural-urban commuting area (RUCA) code based on the most recent publicly available dataset. Rural residence is RUCA code 10 (https://www.ers.usda.gov/data-products/rural-urban-commuting-area-codes/documentation//).
Median Area Deprivation Index (ADI)	National percentile rank based on the most recent publicly available dataset (2021 v4.0.1). Calculated by taking the median ADI national percentile rank among all 9-digit zip code records that contain the patient's 5-digit zip code prefix (https://www.neighborhoodatlas.medicine.wisc.edu/). A higher number indicates a greater level of socioeconomic disadvantage, accounting for factors such as income, education, employment, and housing quality.
Procedure setting	Setting where the procedure was performed (1. hospital outpatient, 2. hospital inpatient, 3. ambulatory center, 4. office, or 5. not reported)
**Comorbidities**	
Smoking status	Current, prior (quit ≥ 1 month ago), or never
Hypertension	Documented in history or recorded blood pressure > 130/80 on 3 or more occasions
Diabetes	Documented in history or receiving anti-hyperglycemic medications including insulin
Coronary artery disease	History of myocardial infarction, stable angina, or unstable angina

© 2024 Li B et al. *JAMA Network Open.*

**Features (n = 75)**	**Definition based on Vascular Quality Initiative Data Dictionary**
Dysrhythmia	Dysrhythmia at the time of treatment as documented in the medical record or electrocardiogram, including atrial fibrillation/flutter, supraventricular arrhythmia, ventricular dysrhythmia, atrioventricular block, pacemaker, implantable cardioverter defibrillator, or other dysrhythmia
Congestive heart failure	Documented in history and severity classified based on the New York Heart Association (NYHA) heart failure classification
Prior stroke	History of major or minor stroke
Chronic obstructive pulmonary disease	Documented in history and whether patient is not treated, on medications, or home oxygen
Dialysis	Patient is currently on hemodialysis or peritoneal dialysis
**Previous procedures**	
Any intervention for peripheral artery disease	Prior bypass, endarterectomy, or endovascular intervention for peripheral artery disease
Suprainguinal bypass or angioplasty/stent	Prior suprainguinal bypass or angioplasty/stent for peripheral artery disease
Infrainguinal bypass or angioplasty/stent	Prior infrainguinal bypass or angioplasty/stent for peripheral artery disease
Lower extremity amputation	Prior major (above ankle) or minor (below ankle) lower extremity amputation
Coronary artery bypass graft	Prior coronary artery bypass graft
Percutaneous coronary intervention	Prior percutaneous coronary intervention
Carotid endarterectomy or stent	Prior carotid endarterectomy or stent
Aortic aneurysm repair	Prior repair of aortic aneurysm
**Functional status**	
Living status	Last living status prior to hospitalization (home, nursing home, or homeless)
Baseline function	Baseline functional status (full, light work, self care, assisted care, or bed bound)
Pre-operative ambulatory status	1. Ambulates independently<br>2. Ambulates with assistance or prosthesis<br>3. Wheelchair-dependent<br>4. Bedridden
**Investigations**	
Creatinine	Most recent pre-operative creatinine within 6 months prior to surgery (umol/L)
Cardiac stress test	Includes stress electrocardiogram, stress echocardiogram, or nuclear stress test within 2 years of surgery and reported as not done, normal, or abnormal (positive for ischemia, positive for infarction, or positive for both ischemia and infarction)

© 2024 Li B et al. *JAMA Network Open.*

Features (n = 75)	Definition based on Vascular Quality Initiative Data Dictionary
**Medications**	Taken within 36 hours of procedure
Acetylsalicylic acid	Includes drugs that contain acetylsalicylic acid
P2Y12 antagonist	Includes clopidogrel, prasugrel, ticlopidine, and ticagrelor
Statin	Includes atorvastatin, fluvastatin, lovastatin, pitavastatin, pravastatin, rosuvastatin, simvastatin, or a combination medication including a statin
Angiotensin converting enzyme inhibitor (ACE-I) or angiotensin II receptor blocker (ARB)	Includes benazepril, captopril, enalapril, fosinopril, lisinopril, moexipril, perindopril, ramipril, and trandolapril
Anticoagulant	Includes warfarin, dabigatran, rivaroxaban, or other anticoagulant
**Anatomy**	
Ankle brachial index	Ankle brachial index of index limb
Number of arteries treated	Total number of arteries treated during procedure
Primary artery treated	Primary artery of interest for treatment<br>1. Aorta<br>2. Common iliac<br>3. External/internal iliac<br>4. Common femoral<br>5. Profunda<br>6. Superficial femoral<br>7. Popliteal<br>8. Tibial<br>9. Pedal
Second artery treated	Second artery treated if applicable
Third artery treated	Third artery treated if applicable
Fourth artery treated	Fourth artery treated if applicable
Primary procedure type	Primary procedure performed on primary artery of interest<br>1. Angioplasty<br>2. Stent<br>3. Atherectomy
Procedure performed on second artery	Primary procedure performed on second artery if applicable
Procedure performed on third artery	Primary procedure performed on third artery if applicable
Procedure performed on fourth artery	Primary procedure performed on fourth artery if applicable
Total treatment length of primary artery	Total length of treatment for primary artery of interest (cm) if applicable
Total treatment length of second artery	Total length of treatment for second artery (cm) if applicable
Total treatment length of third artery	Total length of treatment for third artery (cm) if applicable

© 2024 Li B et al. *JAMA Network Open.*

Features (n = 75)	Definition based on Vascular Quality Initiative Data Dictionary
Total treatment length of fourth artery	Total length of treatment for fourth artery (cm) if applicable
Total occlusion length of primary lesion	Total length of occlusion of primary lesion of interest (cm)
Total occlusion length of second lesion	Total length of occlusion of second lesion (cm) if applicable
Total occlusion length of third lesion	Total length of occlusion of third lesion (cm) if applicable
Total occlusion length of fourth lesion	Total length of occlusion of fourth lesion (cm) if applicable
Calcification of primary lesion	Determined based on fluoroscopy, computed tomography, or intravascular ultrasound on primary lesion of interest<br>1. None (no calcification visible)<br>2. Focal (calcification on one side of the artery less than half the length of the lesion)<br>3. Mild (calcification on one side of the artery greater than half of the length of the lesion)<br>4. Moderate (calcification on both sides of the artery less than half the length of the lesion)<br>5. Severe (calcification on both sides of the artery greater than half of the length of the lesion)<br>6. Not evaluated
Calcification of second lesion	Severity of calcification of second lesion if applicable
Calcification of third lesion	Severity of calcification of third lesion if applicable
Calcification of fourth lesion	Severity of calcification of fourth lesion if applicable
TASC grade of primary lesion	Anatomic grading of primary lesion of interest (A, B, C, or D) determined by the treating physician
TASC grade of second lesion	Anatomic grading of second lesion (A, B, C, or D) determined by the treating physician if applicable
TASC grade of third lesion	Anatomic grading of second lesion (A, B, C, or D) determined by the treating physician if applicable
TASC grade of fourth lesion	Anatomic grading of second lesion (A, B, C, or D) determined by the treating physician if applicable
Side of primary artery treated	Leg on which the primary artery was treated if applicable
Side of second artery treated	Leg on which the second artery was treated if applicable
Side of third artery treated	Leg on which the third artery was treated if applicable
Side of fourth artery treated	Leg on which the fourth artery was treated if applicable

© 2024 Li B et al. *JAMA Network Open.*

Features (n = 75)	Definition based on Vascular Quality Initiative Data Dictionary
**Other pre-procedural characteristics**	
Symptom status	Asymptomatic, claudication, or chronic limb threatening ischemia (rest pain or tissue loss)
Treatment performed to maintain existing bypass patency	The patient received treatment to maintain patency of an existing bypass
Urgency	Elective [planned/scheduled procedure], urgent [surgery within 24 hours of admission], or emergent [surgery within 6 hours of admission]

* Abbreviation: TASC (Trans-Atlantic Society Consensus).

© 2024 Li B et al. *JAMA Network Open.*

eTable 2. Intraoperative Features for Machine Learning Models

Features (n = 24)	Definition based on Vascular Quality Initiative Data Dictionary
Access artery	Artery used to obtain vascular access<br>1. Femoral<br>2. Popliteal<br>3. Pedal<br>4. Axillary<br>5. Brachial<br>6. Radial<br>7. Graft<br>8. Other
Access side	Side of leg or arm used to obtain vascular access if applicable
Access guidance	Imaging guidance used to obtain vascular access<br>1. None<br>2. Fluoroscopy<br>3. Ultrasound<br>4. Open exposure without endarterectomy<br>5. Open exposure with endarterectomy<br>6. Not reported
Largest sheath used	Largest size of sheath used during procedure in French
Number of devices used to treat primary artery	Number of devices (balloons, stents, atherectomy devices, etc.) used to treat primary artery of interest
Number of devices used to treat second artery	Number of devices (balloons, stents, atherectomy devices, etc.) used to treat second artery if applicable
Number of devices used to treat third artery	Number of devices (balloons, stents, atherectomy devices, etc.) used to treat third artery if applicable
Number of devices used to treat fourth artery	Number of devices (balloons, stents, atherectomy devices, etc.) used to treat fourth artery if applicable
Procedural anticoagulant	Anticoagulant administered during the procedure<br>1. None<br>2. Heparin<br>3. Bivalirudin<br>4. Other
Protamine	Protamine administered at the end of the procedure
Adjunctive procedures	Adjunctive procedures performed during intervention
Pharmacologic thrombolysis	Intra-arterial infusion of thrombolytic agents such as tissue plasminogen activator or alteplase
Mechanical thrombolysis	Use of rotational, rheolytic, or ultrasound device to treat lesion
Embolic protection device	Embolic protection device inserted distal to the lesion to prevent distal embolization
Intravascular ultrasound	Intravascular ultrasound used during procedure

Features (n = 24)	Definition based on Vascular Quality Initiative Data Dictionary
Chronic total occlusion device	Chronic total occlusion device used to cross a chronic total occlusion
Suction thrombectomy	Device used for suction or aspiration of thrombus
Femoral endarterectomy	Concomitant endarterectomy of the femoral artery
Carbon dioxide angiography	Carbon dioxide angiography performed during procedure
Total contrast volume	Total full-strength equivalent of contrast administered during procedure (ml)
Total fluoroscopy time	Total fluoroscopy time in minutes
Technical success for primary lesion	Residual stenosis of primary lesion ≤ 30% at the end of the procedure
Technical success for second lesion	Residual stenosis of second lesion ≤ 30% at the end of the procedure if applicable
Technical success for third lesion	Residual stenosis of third lesion ≤ 30% at the end of the procedure if applicable
Technical success for fourth lesion	Residual stenosis of fourth lesion ≤ 30% at the end of the procedure if applicable

© 2024 Li B et al. *JAMA Network Open.*

eTable 3. Postoperative Features for Machine Learning Models

**Features (n = 13)**	**Definition based on Vascular Quality Initiative Data Dictionary**
In-hospital cardiac complication	New dysrhythmia, myocardial infarction, congestive heart failure, or other clinically significant cardiac complication prior to discharge
In-hospital myocardial infarction	Combination of clinical symptoms [i.e. chest pain/dyspnea], electrocardiogram changes, and troponin elevation prior to discharge
In-hospital pulmonary complication	Pneumonia, need for ventilator support, or other clinically significant pulmonary complication such as pulmonary embolism prior to discharge
In-hospital renal complication	New increase in creatinine ≥ 0.5mg/dl (44.2 umol/L) or new dialysis (peritoneal, hemodialysis or hemo-filtration) prior to discharge
In-hospital access site complication	Hematoma, stenosis/occlusion, infection, pseudoaneurysm, or arteriovenous fistula secondary to vascular access prior to discharge
In-hospital contrast complication	Allergic reaction, arrhythmia, seizure, or hypertensive emergency secondary to contrast administration prior to discharge
Length of stay	Time from admission to discharge (days)
Discharge medications	Prescribed at discharge
Acetylsalicylic acid	Includes drugs that contain acetylsalicylic acid
P2Y12 antagonist	Includes clopidogrel, prasugrel, ticlopidine, and ticagrelor
Statin	Includes atorvastatin, fluvastatin, lovastatin, pitavastatin, pravastatin, rosuvastatin, simvastatin, or a combination medication including a statin
Angiotensin converting enzyme inhibitor (ACE-I) or angiotensin II receptor blocker (ARB)	Includes benazepril, captopril, enalapril, fosinopril, lisinopril, moexipril, perindopril, ramipril, and trandolapril
Anticoagulant	Includes warfarin, dabigatran, rivaroxaban, or other anticoagulant
Non-home discharge	Discharge to nursing home, rehabilitation unit, or other hospital

© 2024 Li B et al. *JAMA Network Open.*

eTable 4. Selection of Extreme Gradient Boosting (XGBoost) Model Hyperparameters Using Grid Search and Cross-Validation

Hyperparameter	Values tested through grid search and cross validation*	Optimal value chosen to maximize AUROC
Number of rounds	50, 100, 150, 200, 250, 300, 350, 400, 450, 500	200
Maximum tree depth	2, 3, 4, 5, 6, 7, 8, 9	3
Learning rate	0.4, 0.3, 0.2, 0.1, 0.05, 0.01, 0.001	0.3
Gamma	0, 0.1, 1, 1.5, 2	0
Column sample by tree	0.5, 0.6, 0.7, 0.8, 0.9, 1	0.6
Minimum child weight	1, 3, 5, 7, 10	1
Subsample	0.5, 0.6, 0.7, 0.8, 0.9, 1	0.9

*Grid search and cross validation are exhaustive methods that iteratively train and evaluate models using every combination of specified hyperparameter values and selects the set of hyperparameter values that optimize model performance.

Abbreviation: AUROC (area under the receiver operating characteristic curve).

© 2024 Li B et al. *JAMA Network Open.*

eTable 5. Preoperative Demographic and Clinical Characteristics of Patients Undergoing Endovascular Intervention for Peripheral Artery Disease With and Without Major Adverse Limb Event or Death at 1 Year

	**Absence of MALE or death at 1 year (n = 163,994)**	**Presence of MALE or death at 1 year (n = 71,683)**	**P**
**Demographics**			
Age, years, mean (SD)	68.0 (10.9)	69.3 (11.7)	< 0.001
Sex			
Female	65,426 (39.9)	29,553 (41.2)	< 0.001
Male	98,568 (60.1)	42,130 (58.8)	
BMI, kg/m^2, mean (SD)	28.1 (6.2)	27.5 (6.4)	< 0.001
Race*			
American Indian or Alaskan Native	697 (0.4)	451 (0.6)	< 0.001
Asian	2,696 (1.6)	1,757 (2.5)	
Black	24,701 (15.1)	13,224 (18.4)	
Native Hawaiian or other Pacific Islander	163 (0.1)	101 (0.1)	
White	128,047 (78.1)	52,691 (73.5)	
More than 1 race	346 (0.2)	193 (0.3)	
Unknown/other^	7,344 (4.5)	3,266 (4.6)	
Hispanic ethnicity*	8,688 (5.3)	4,688 (6.5)	< 0.001
Insurance status			
Medicare	76,017 (46.4)	36,493 (50.9)	< 0.001
Medicaid	11,245 (6.9)	4,974 (6.9)	
Commercial	50,378 (30.7)	17,917 (25.0)	
Medicare Advantage	7,627 (4.7)	4,117 (5.7)	
Military/Veterans Affairs	1,682 (1.0)	658 (0.9)	
Non-US Insurance	4,040 (2.5)	2,227 (3.1)	
Self-pay (uninsured)	2,507 (1.5)	1,022 (1.4)	
Unknown/other	10,498 (6.4)	4,275 (6.0)	
Rural residence	5,304 (3.2)	2,306 (3.2)	0.84
Area Deprivation Index percentile, median (IQR)	56 (37 – 75)	57 (38 – 76)	< 0.001
Procedure setting			
Hospital outpatient	62,075 (37.9)	20,767 (29.0)	< 0.001
Hospital inpatient	41,784 (25.5)	26,875 (37.5)	
Ambulatory center	3,199 (2.0)	1,253 (1.8)	
Office	4,130 (2.5)	1,251 (1.8)	
Other/not reported	52,806 (32.2)	21,537 (30.0)	
**Comorbidities**			
Smoking status			
Never	32,385 (19.7)	19,020 (26.5)	< 0.001

© 2024 Li B et al. *JAMA Network Open.*

	Absence of MALE or death at 1 year (n = 163,994)	Presence of MALE or death at 1 year (n = 71,683)	P
Prior	73,699 (44.9)	32,020 (44.7)	
Current	57,910 (35.3)	20,643 (28.8)	
Hypertension	144,225 (87.9)	64,582 (90.1)	< 0.001
Diabetes	83,138 (50.7)	45,277 (63.2)	< 0.001
Coronary artery disease	52,745 (32.2)	26,513 (37.0)	< 0.001
Dysrhythmia	19,826 (12.1)	13,565 (18.9)	< 0.001
Congestive heart failure	27,371 (16.7)	20,753 (29.0)	< 0.001
Prior stroke	15,989 (9.7)	9,564 (13.3)	< 0.001
Chronic obstructive pulmonary disease	41,806 (25.5)	19,463 (27.2)	< 0.001
Dialysis	8,257 (5.0)	11,884 (16.6)	< 0.001
**Previous procedures**			
Any intervention for peripheral artery disease	80,067 (48.8)	40,080 (55.9)	< 0.001
Suprainguinal bypass or angioplasty/stent	32,358 (19.7)	13,945 (19.5)	0.001
Infrainguinal bypass or angioplasty/stent	64,420 (39.3)	34,991 (48.8)	< 0.001
Lower extremity amputation			
Minor	17,096 (10.4)	13,775 (19.2)	< 0.001
Major	4,672 (2.9)	4,541 (6.3)	
Coronary artery bypass graft	29,823 (18.2)	15,220 (21.2)	< 0.001
Percutaneous coronary intervention	36,840 (22.5)	17,149 (24.0)	< 0.001
Carotid endarterectomy or stent	12,958 (7.9)	5,463 (7.6)	< 0.001
Aortic aneurysm repair	3,449 (2.1)	1,270 (1.8)	< 0.001
**Functional status**			
Living status			
Home	159,665 (97.4)	67,158 (93.7)	< 0.001
Nursing home	4,011 (2.5)	4,389 (6.1)	
Homeless	318 (0.2)	136 (0.2)	
Baseline function			
Full	109,059 (66.5)	40,041 (55.9)	< 0.001
Light work	29,390 (17.9)	12,840 (17.9)	
Self care	18,359 (11.2)	11,380 (15.9)	
Assisted care	6,784 (4.1)	6,899 (9.6)	
Bed bound	402 (0.2)	523 (0.7)	
Pre-operative ambulatory status			
Independent	126,544 (77.2)	42,063 (58.7)	< 0.001
With assistance or prosthesis	29,909 (18.2)	21,540 (30.0)	
Wheelchair-dependent	7,109 (4.3)	7,521 (10.5)	
Bedridden	432 (0.3)	559 (0.8)	

© 2024 Li B et al. *JAMA Network Open.*

	Absence of MALE or death at 1 year (n = 163,994)	Presence of MALE or death at 1 year (n = 71,683)	P
**Investigations**			
Creatinine, umol/L, mean (SD)	96.3 (23.3)	101.0 (51.8)	< 0.001
Cardiac stress test			
Not done	132,041 (80.5)	58,557 (81.7)	< 0.001
Normal	22,632 (13.8)	8,498 (11.9)	
Abnormal	9,321 (5.7)	4,628 (6.5)	
**Medications**			
Acetylsalicylic acid	120,023 (73.2)	50,031 (69.8)	< 0.001
P2Y12 antagonist	70,242 (42.8)	31,241 (43.6)	< 0.001
Statin	123,319 (75.2)	52,441 (73.2)	< 0.001
ACE-I/ARB	84,823 (51.7)	33,380 (46.6)	< 0.001
Anticoagulant	25,901 (15.8)	16,663 (23.2)	< 0.001
**Anatomy**			
Ankle brachial index, median (IQR)	0.73 (0.53 – 0.85)	0.80 (0.50 – 0.85)	< 0.001
Number of arteries treated, median (IQR)	1 (1 – 2)	2 (1 – 2)	< 0.001
Primary artery treated			
Aorta	1,839 (1.1)	343 (0.5)	< 0.001
Common iliac	29,491 (18.0)	6,750 (9.4)	
External/internal iliac	20,198 (12.3)	6,445 (9.0)	
Common femoral	5,106 (3.1)	2,222 (3.1)	
Profunda	1,002 (0.6)	516 (0.7)	
Superficial femoral	69,375 (42.3)	32,049 (44.7)	
Popliteal	14,633 (8.9)	7,902 (11.0)	
Tibial	21,984 (13.4)	15,167 (21.2)	
Pedal	366 (0.2)	289 (0.4)	
Procedure type			
Angioplasty	96,810 (59.0)	46,487 (64.9)	< 0.001
Stent	42,565 (26.0)	14,295 (19.9)	
Atherectomy	24,619 (15.0)	10,901 (15.2)	
Total treatment length, cm, median (IQR)	10 (4 – 15)	13 (6 – 16)	< 0.001
Total occlusion length, cm, median (IQR)	4 (0 – 6)	5 (0 – 6)	< 0.001
Calcification			
None	17,465 (10.6)	7,238 (10.1)	< 0.001
Focal	5,075 (3.1)	1,961 (2.7)	
Mild	7,878 (4.8)	3,207 (4.5)	
Moderate	16,463 (10.0)	6,920 (9.7)	
Severe	24,572 (15.0)	11,933 (16.6)	
Not evaluated	92,541 (56.4)	40,424 (56.4)	

© 2024 Li B et al. *JAMA Network Open.*

	**Absence of MALE or death at 1 year (n = 163,994)**	**Presence of MALE or death at 1 year (n = 71,683)**	**P**
TASC grade			
A	34,714 (21.2)	10,092 (14.1)	< 0.001
B	33,409 (20.4)	12,345 (17.2)	
C	28,125 (17.2)	13,829 (19.3)	
D	21,239 (13.0)	14,097 (19.7)	
Not reported	46,507 (28.4)	21,320 (29.7)	
**Indications/urgency**			
Symptom status			
Asymptomatic	7,822 (4.8)	3,567 (5.0)	< 0.001
Claudication	81,530 (49.7)	17,301 (24.1)	
Chronic limb threatening ischemia			
Rest pain	23,389 (14.3)	11,595 (16.2)	
Tissue loss	51,253 (31.3)	39,220 (54.7)	
Treatment performed to maintain existing bypass patency	2,241 (1.4)	1,017 (1.4)	0.33
Urgency			
Elective	143,897 (87.7)	56,407 (78.7)	< 0.001
Urgent	17,903 (10.9)	13,562 (18.9)	
Emergent	2,914 (1.3)	1,714 (2.4)	

Values are reported as No. (%) unless otherwise indicated.
*Race and ethnicity were identified through participant self-report and the source of the classifications used was based on the U.S. Census Bureau.
^Unknown/other race refers to unknown race of the participant or a race reported by the participant that is not categorized as one of the following based on the U.S. Census Bureau: American Indian or Alaskan Native; Asian; Black; Native Hawaiian or other Pacific Islander; or White.
Abbreviations: BMI (body mass index), ACE-I (angiotensin converting enzyme inhibitor), ARB (angiotensin II receptor blocker), TASC (Trans-Atlantic Society Consensus), SD (standard deviation), IQR (interquartile range).

© 2024 Li B et al. *JAMA Network Open.*

eTable 6. Intraoperative Characteristics of Patients Undergoing Endovascular Intervention for Peripheral Artery Disease With and Without Major Adverse Limb Event or Death at 1 Year

	**Absence of MALE or death at 1 year (n = 163,994)**	**Presence of MALE or death at 1 year (n = 71,683)**	**P**
Access artery			
Femoral	156,955 (95.7)	68,439 (95.5)	< 0.001
Popliteal	407 (0.2)	238 (0.3)	
Pedal	826 (0.5)	515 (0.7)	
Axillary	879 (0.5)	376 (0.5)	
Brachial	1,788 (1.1)	839 (1.2)	
Radial	1,110 (0.7)	336 (0.5)	
Graft	747 (0.5)	473 (0.7)	
Other	1,282 (0.8)	451 (0.6)	
Access guidance			
None	19,692 (12.0)	7,542 (10.5)	< 0.001
Fluoroscopy	21,547 (13.1)	7,958 (11.1)	
Ultrasound	108,753 (66.3)	51,064 (71.2)	
Open exposure without endarterectomy	4,917 (3.0)	1,905 (2.7)	
Open exposure with endarterectomy	7,710 (4.7)	2,757 (3.9)	
Not reported	1,375 (0.8)	457 (0.6)	
Procedural anticoagulant			
None	58,273 (35.5)	23,904 (33.3)	< 0.001
Heparin	104,460 (63.7)	47,251 (65.9)	
Bivalirudin	1,135 (0.7)	431 (0.6)	
Other	126 (0.08)	97 (0.1)	
Protamine	39,535 (24.1)	16,726 (23.3)	< 0.001
Adjunctive procedures			
Pharmacologic thrombolysis	2,476 (1.5)	1,805 (2.5)	< 0.001
Mechanical thrombolysis	1,566 (1.0)	1,141 (1.6)	< 0.001
Embolic protection device	6,651 (4.1)	2,920 (4.1)	0.85
Intravascular ultrasound	6,488 (4.0)	2,629 (3.7)	< 0.001
Chronic total occlusion device	2,351 (1.4)	1,193 (1.7)	< 0.001
Suction thrombectomy	994 (0.6)	806 (1.1)	< 0.001
Femoral endarterectomy	7,909 (4.8)	3,975 (5.6)	< 0.001
Carbon dioxide angiography	6,004 (3.7)	3,612 (5.0)	< 0.001
Total contrast volume, ml, median (IQR)	80 (49 – 120)	76 (48 – 115)	< 0.001
Total fluoroscopy time, min, median (IQR)	14.7 (8.6 – 21.7)	18.0 (10.5 – 26.0)	< 0.001
Technical success (residual stenosis ≤ 30%)	160,162 (97.7)	68,474 (95.5)	< 0.001

© 2024 Li B et al. *JAMA Network Open.*

Values are reported as No. (%) unless otherwise indicated.
Abbreviations: IQR (interquartile range).

© 2024 Li B et al. *JAMA Network Open.*

eTable 7. Postoperative In-Hospital Characteristics and Complications of Patients Undergoing Endovascular Intervention for Peripheral Artery Disease With and Without Major Adverse Limb Event or Death at 1 Year

	**Absence of MALE or death at 1 year (n = 163,994)**	**Presence of MALE or death at 1 year (n = 71,683)**	**P**
Cardiac complication	1,050 (0.6)	922 (1.3)	< 0.001
Myocardial infarction	159 (0.1)	145 (0.2)	< 0.001
Pulmonary complication	337 (0.2)	397 (0.6)	< 0.001
Renal complication	613 (0.4)	696 (1.0)	< 0.001
Access site complication	2,659 (1.6)	1,463 (2.0)	< 0.001
Contrast complication	28 (0.02)	18 (0.03)	0.26
Length of stay, days, median (IQR)	0 (0 – 22)	1 (0 – 26)	< 0.001
Discharge medications			
Acetylsalicylic acid	132,273 (80.7)	54,965 (76.7)	< 0.001
P2Y12 antagonist	122,961 (75.0)	50,773 (70.8)	< 0.001
Statin	131,865 (80.4)	56,352 (78.6)	< 0.001
ACE-I/ARB	78,034 (47.6)	30,274 (42.2)	< 0.001
Anticoagulant	31,029 (18.9)	19,995 (27.9)	< 0.001
Non-home discharge	12,246 (7.5)	12,784 (17.8)	< 0.001

Values are reported as No. (%) unless otherwise indicated.
Abbreviations: ACE-I (angiotensin converting enzyme inhibitor), ARB (angiotensin II receptor blocker), IQR (interquartile range).

© 2024 Li B et al. *JAMA Network Open.*

eTable 8. XGBoost Performance on Test Set Data for Predicting 1-Year Primary and Secondary Outcomes Following Endovascular Intervention for Peripheral Artery Disease at the Preoperative, Intraoperative, and Postoperative Stages

	AUROC (95% CI)	Accuracy (95% CI)	Sensitivity	Specificity	PPV	NPV
**Major adverse limb event or death (primary outcome)**						
Pre-op	0.94 (0.93 – 0.95)	0.86 (0.85 – 0.87)	0.87	0.85	0.85	0.87
Intra-op	0.94 (0.93 – 0.95)	0.87 (0.86 – 0.88)	0.87	0.87	0.87	0.87
Post-op	0.98 (0.97 – 0.99)	0.93 (0.92 – 0.94)	0.93	0.93	0.93	0.93
**Thrombectomy or thrombolysis**						
Pre-op	0.93 (0.92 – 0.94)	0.84 (0.83 – 0.86)	0.87	0.82	0.81	0.88
Intra-op	0.93 (0.92 – 0.94)	0.85 (0.84 – 0.86)	0.88	0.82	0.82	0.88
Post-op	0.97 (0.96 – 0.98)	0.92 (0.91 – 0.93)	0.92	0.92	0.92	0.91
**Surgical reintervention**						
Pre-op	0.91 (0.90 – 0.92)	0.84 (0.83 – 0.85)	0.86	0.82	0.81	0.87
Intra-op	0.93 (0.92 – 0.94)	0.86 (0.85 – 0.87)	0.88	0.84	0.83	0.89
Post-op	0.95 (0.94 – 0.96)	0.89 (0.87 – 0.90)	0.90	0.87	0.87	0.90
**Major amputation**						
Pre-op	0.92 (0.91 – 0.93)	0.84 (0.83 – 0.85)	0.85	0.83	0.84	0.85
Intra-op	0.93 (0.92 – 0.93)	0.85 (0.84 – 0.86)	0.86	0.84	0.84	0.86
Post-op	0.95 (0.94 – 0.95)	0.86 (0.85 – 0.87)	0.86	0.86	0.86	0.86
**Mortality**						
Pre-op	0.94 (0.93 – 0.95)	0.86 (0.85 – 0.88)	0.86	0.87	0.87	0.86
Intra-op	0.95 (0.94 – 0.96)	0.88 (0.86 – 0.89)	0.88	0.87	0.88	0.88
Post-op	0.98 (0.97 – 0.99)	0.93 (0.92 – 0.93)	0.92	0.93	0.93	0.92

*Major adverse limb event defined as a composite of thrombectomy or thrombolysis, surgical reintervention, or major amputation.

Abbreviations: XGBoost (Extreme Gradient Boosting), AUROC (area under the receiver operating characteristic curve), CI (confidence interval), PPV (positive predictive value), NPV (negative predictive value), pre-op (pre-operative), intra-op (intra-operative), post-op (post-operative).

© 2024 Li B et al. *JAMA Network Open.*

eFigure 1. Variables Considered at the Preoperative, Intraoperative, and Postoperative Stages for Model Development

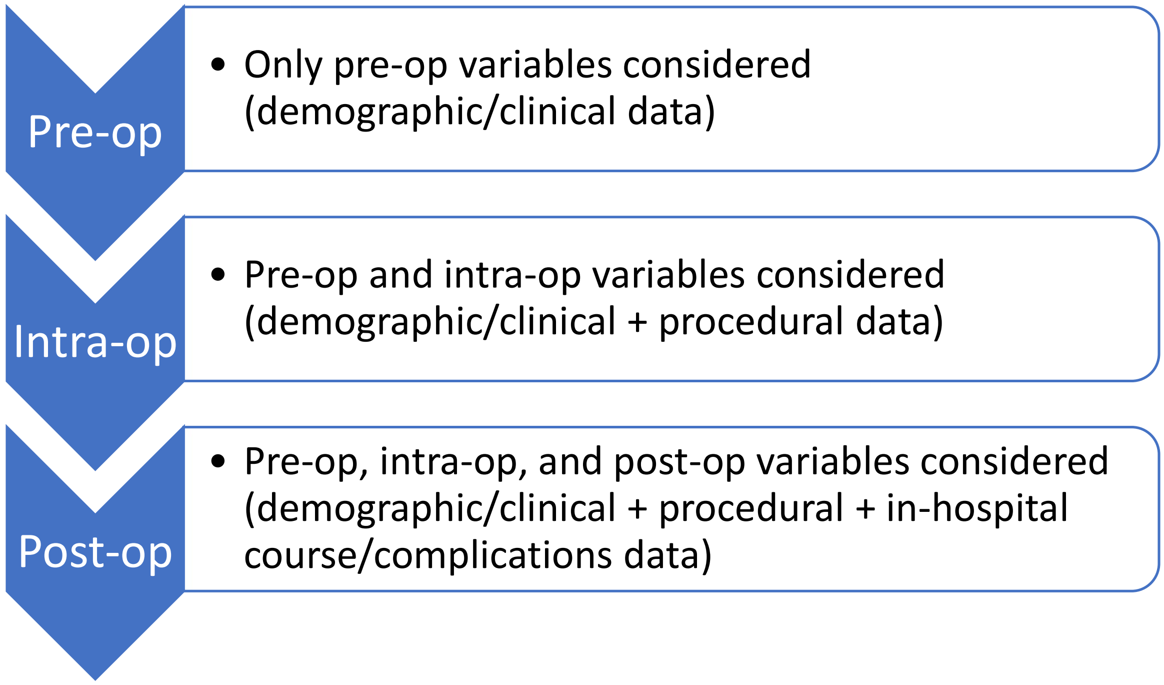

© 2024 Li B et al. *JAMA Network Open.*

eFigure 2. Variable Importance Scores (Gain) for the Top 10 Predictors of 1-Year Major Adverse Limb Event or Death Following Endovascular Intervention for Peripheral Artery Disease in the Extreme Gradient Boosting (XGBoost) Model at the Postoperative Stage With Subgroup Analysis Based on Symptom Status

A) Chronic limb threatening ischemia

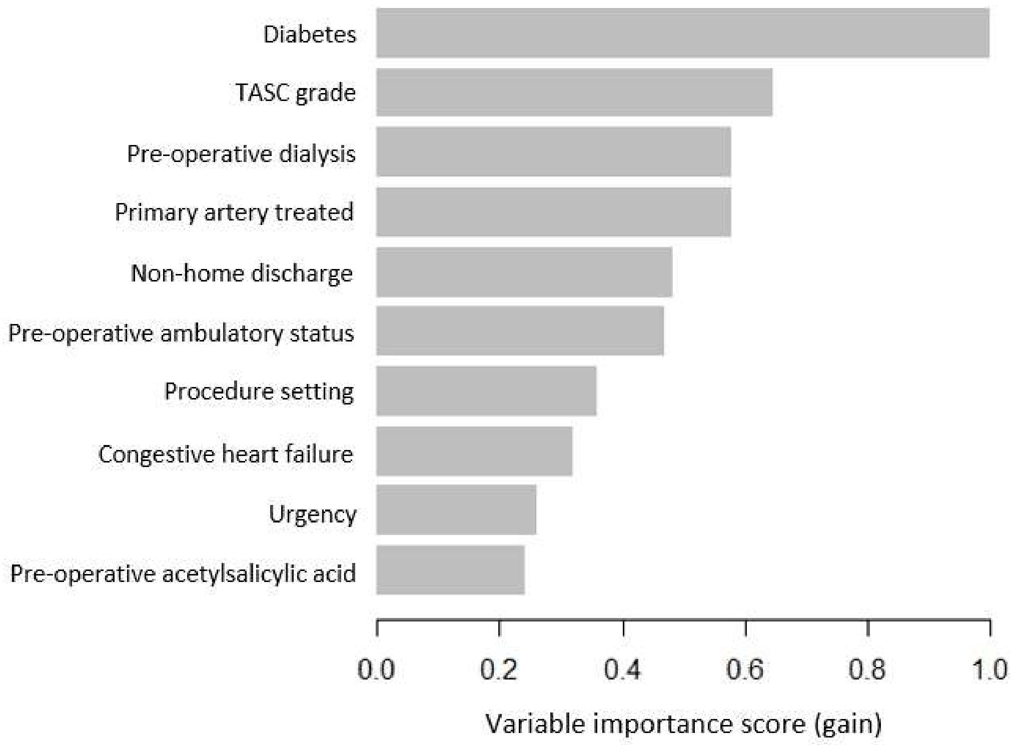

© 2024 Li B et al. *JAMA Network Open.*

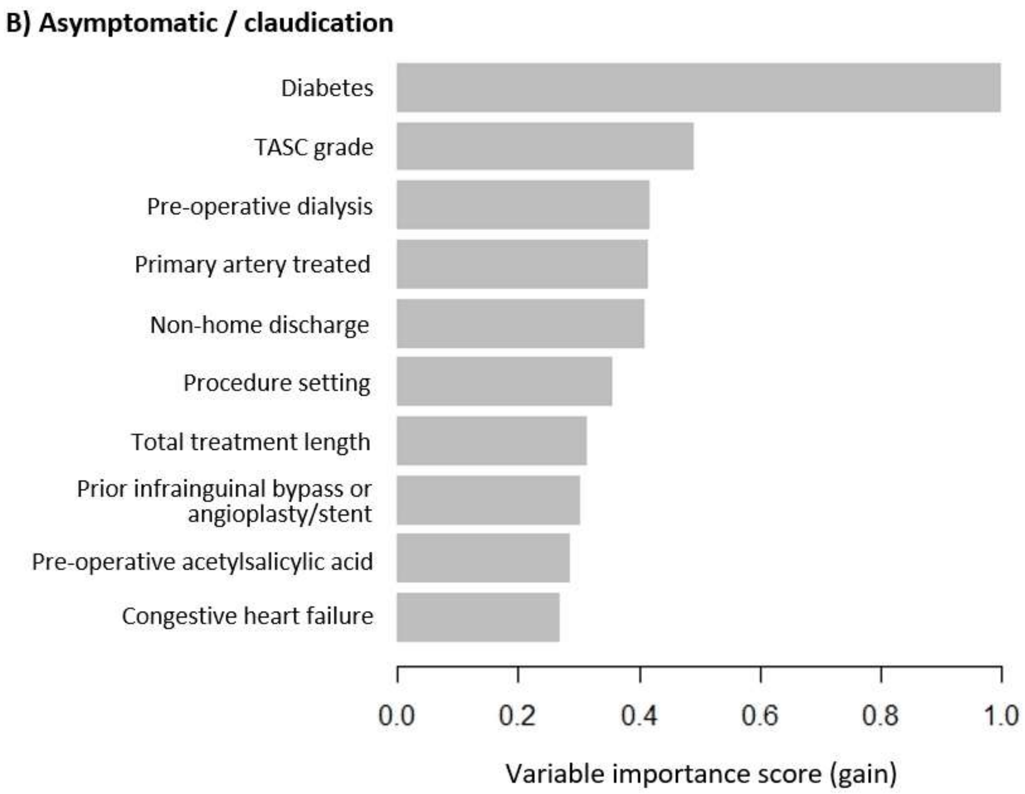

A) chronic limb threatening ischemia and B) asymptomatic/claudication. Abbreviations: TASC (Trans-Atlantic Society Consensus).

© 2024 Li B et al. *JAMA Network Open.*

eFigure 3. Receiver Operating Characteristic Curve for Predicting 1-Year Major Adverse Limb Event or Death Following Endovascular Intervention for Peripheral Artery Disease Using Extreme Gradient Boosting (XGBoost) Model at the Preoperative Stage With Subgroup Analysis Based on Age

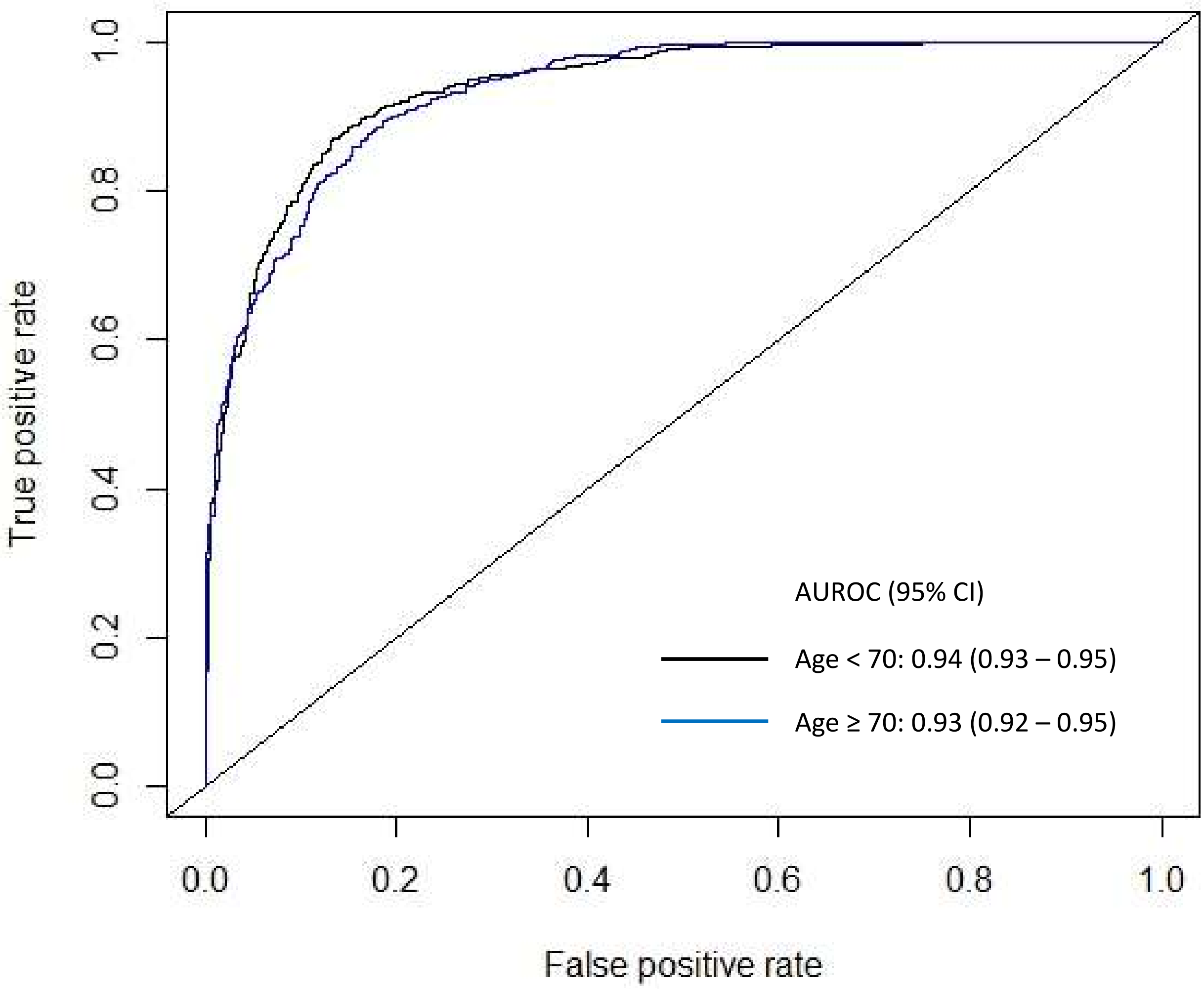

AUROC (area under the receiver operating characteristic curve), CI (confidence interval).

© 2024 Li B et al. *JAMA Network Open.*

eFigure 4. Receiver Operating Characteristic Curve for Predicting 1-Year Major Adverse Limb Event or Death Following Endovascular Intervention for Peripheral Artery Disease Using Extreme Gradient Boosting (XGBoost) Model at the Preoperative Stage With Subgroup Analysis Based on Sex

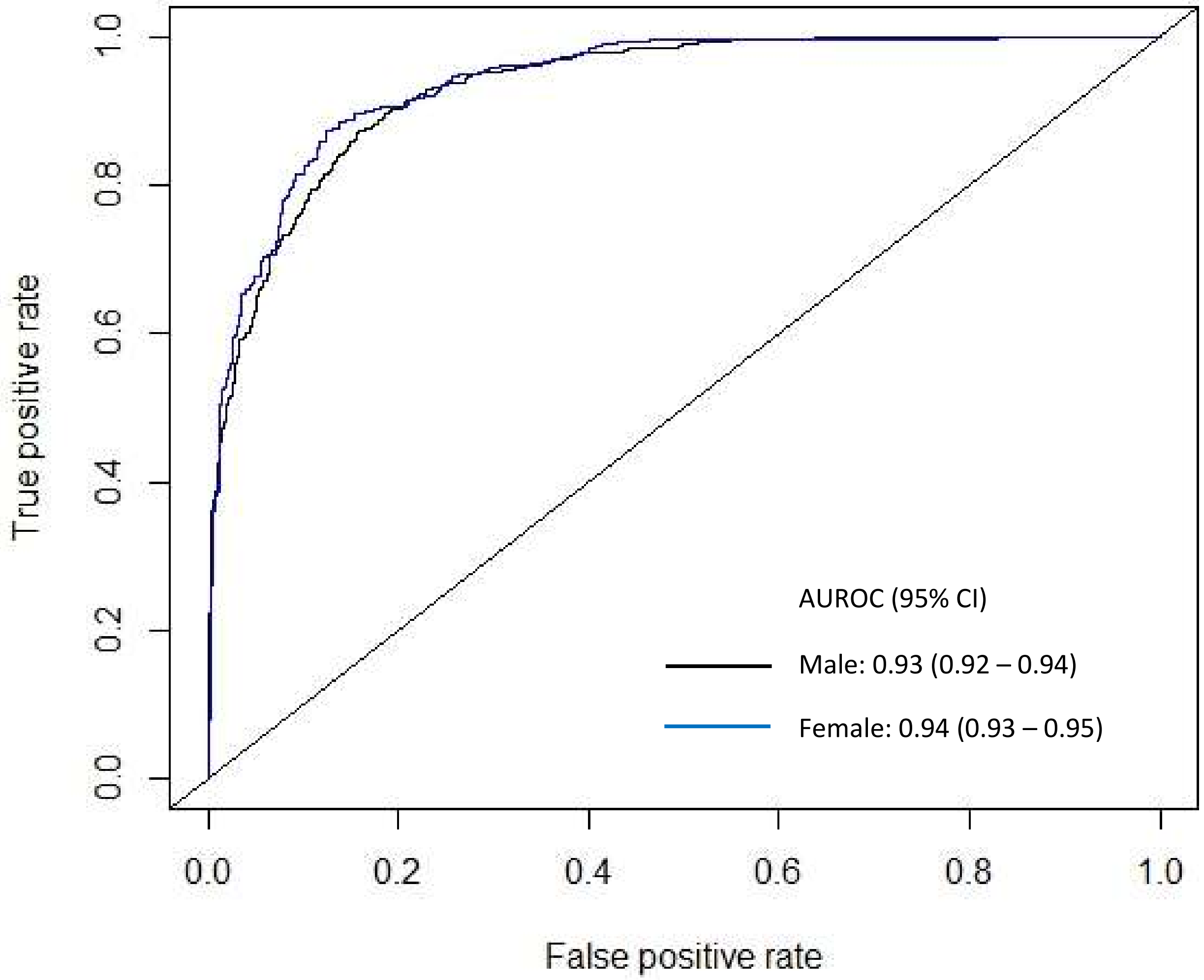

AUROC (area under the receiver operating characteristic curve), CI (confidence interval).

© 2024 Li B et al. *JAMA Network Open.*

eFigure 5. Receiver Operating Characteristic Curve for Predicting 1-Year Major Adverse Limb Event or Death Following Endovascular Intervention for Peripheral Artery Disease Using Extreme Gradient Boosting (XGBoost) Model at the Preoperative Stage With Subgroup Analysis Based on Race

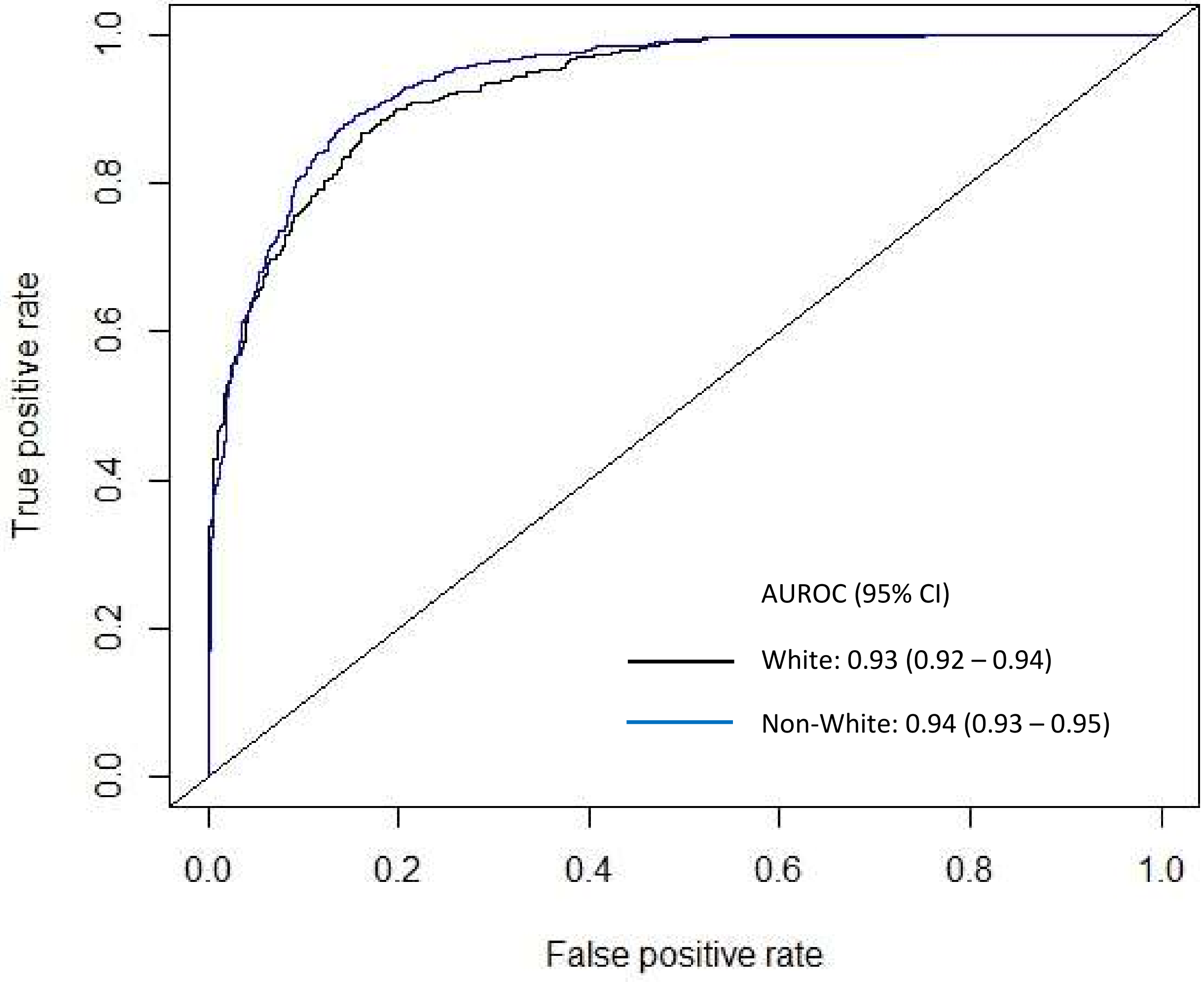

AUROC (area under the receiver operating characteristic curve), CI (confidence interval).

© 2024 Li B et al. *JAMA Network Open.*

eFigure 6. Receiver Operating Characteristic Curve for Predicting 1-Year Major Adverse Limb Event or Death Following Endovascular Intervention for Peripheral Artery Disease Using Extreme Gradient Boosting (XGBoost) Model at the Preoperative Stage With Subgroup Analysis Based on Ethnicity

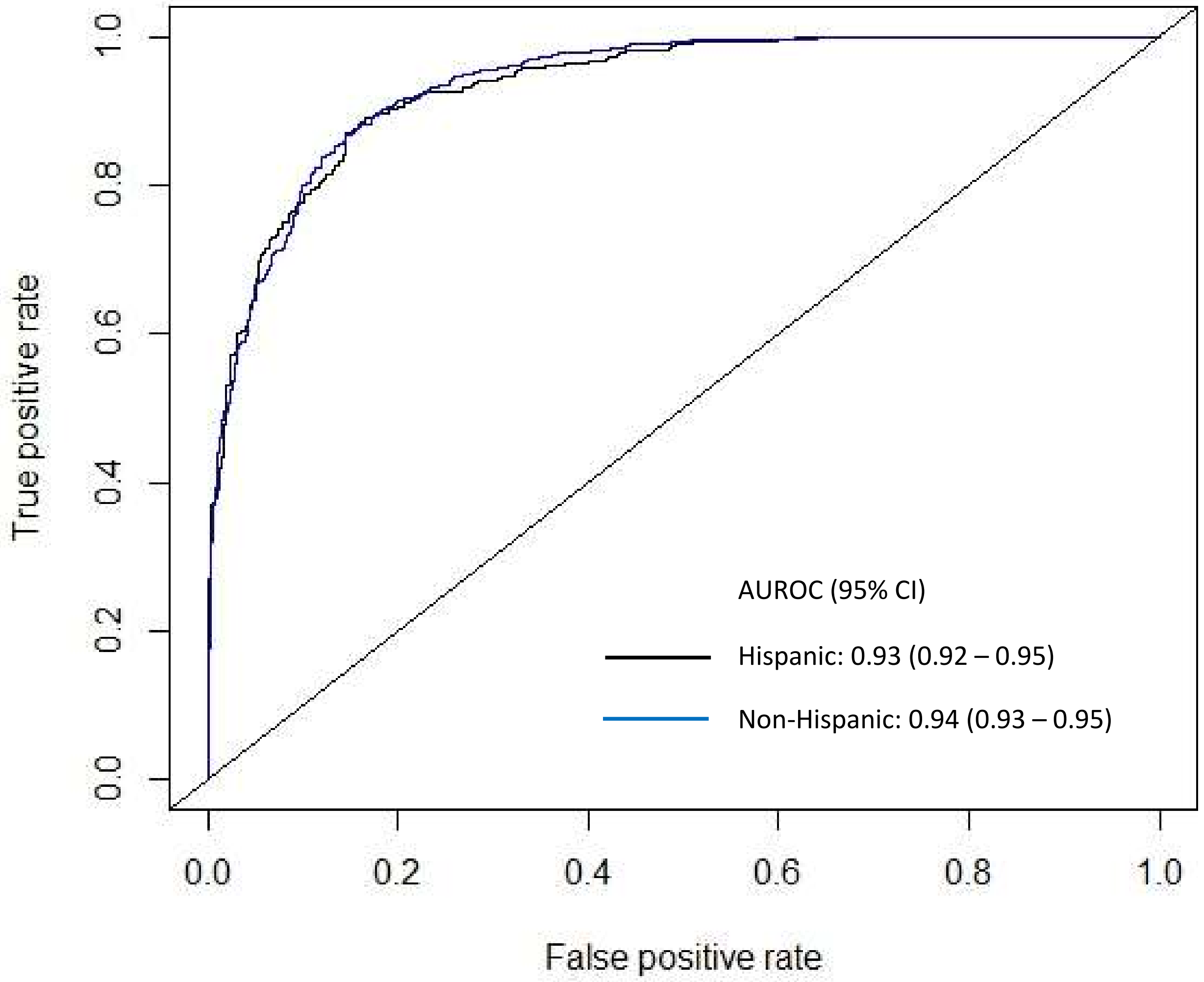

AUROC (area under the receiver operating characteristic curve), CI (confidence interval).

© 2024 Li B et al. *JAMA Network Open.*

eFigure 7. Receiver Operating Characteristic Curve for Predicting 1-Year Major Adverse Limb Event or Death Following Endovascular Intervention for Peripheral Artery Disease Using Extreme Gradient Boosting (XGBoost) Model at the Preoperative Stage With Subgroup Analysis Based on Rurality of Residence

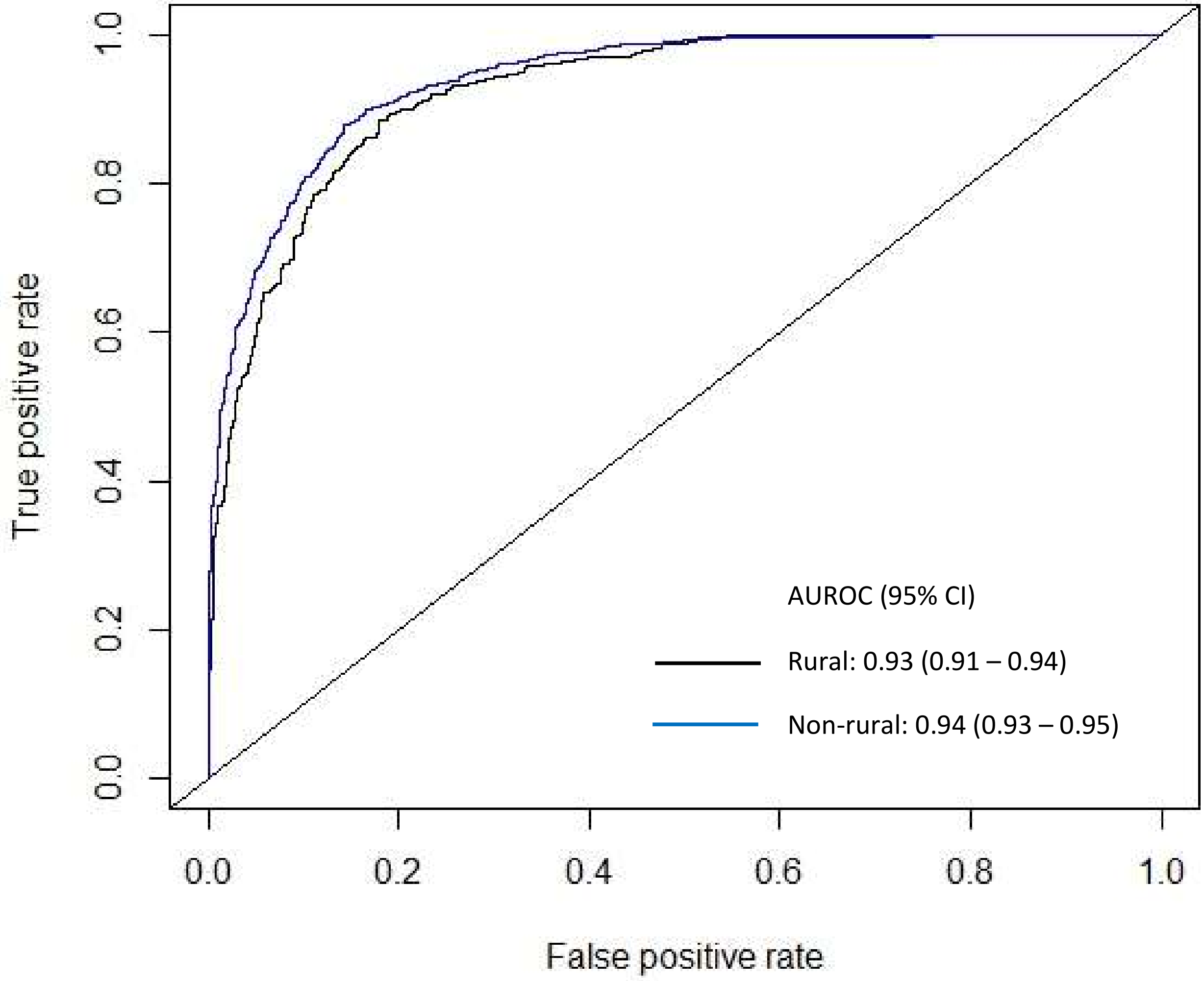

AUROC (area under the receiver operating characteristic curve), CI (confidence interval).

© 2024 Li B et al. *JAMA Network Open.*

eFigure 8. Receiver Operating Characteristic Curve for Predicting 1-Year Major Adverse Limb Event or Death Following Endovascular Intervention for Peripheral Artery Disease Using Extreme Gradient Boosting (XGBoost) Model at the Preoperative Stage With Subgroup Analysis Based on Median Area Deprivation Index (ADI) Percentile

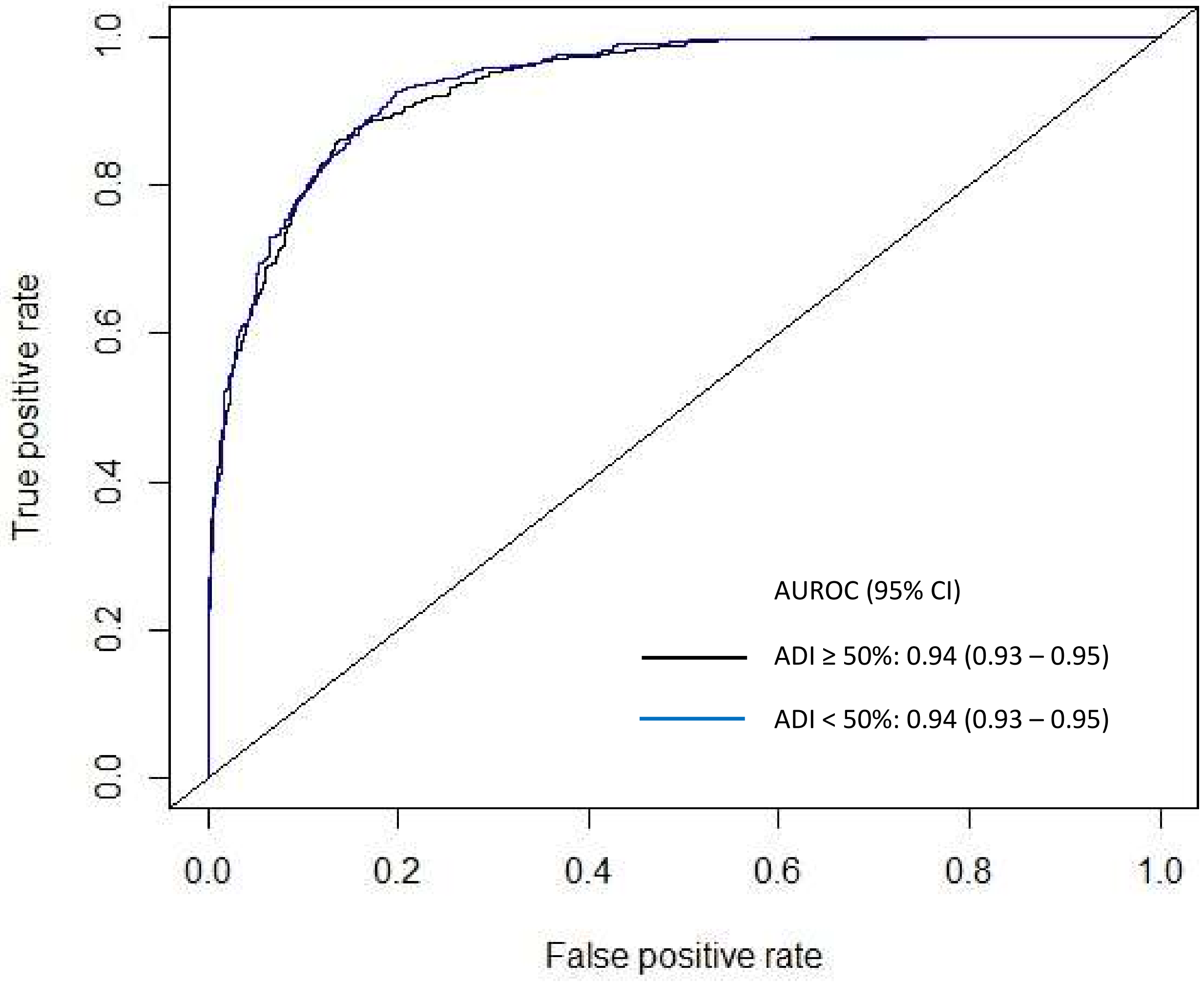

AUROC (area under the receiver operating characteristic curve), CI (confidence interval).

© 2024 Li B et al. *JAMA Network Open.*

eFigure 9. Receiver Operating Characteristic Curve for Predicting 1-Year Major Adverse Limb Event or Death Following Endovascular Intervention for Peripheral Artery Disease Using Extreme Gradient Boosting (XGBoost) Model at the Preoperative Stage With Subgroup Analysis Based on Symptom Status

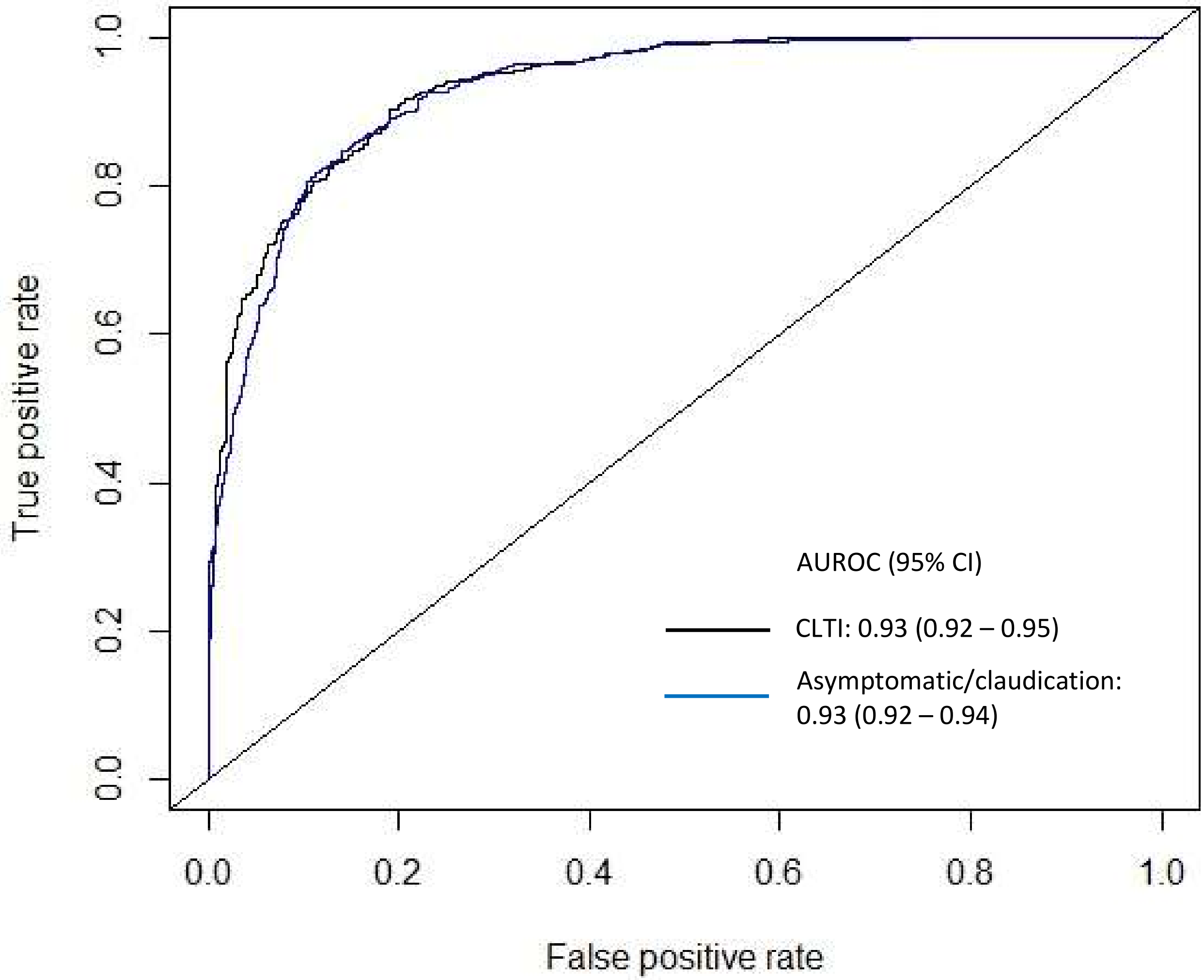

AUROC (area under the receiver operating characteristic curve), CI (confidence interval), CLTI (chronic limb threatening ischemia).

© 2024 Li B et al. *JAMA Network Open.*

eFigure 10. Receiver Operating Characteristic Curve for Predicting 1-Year Major Adverse Limb Event or Death Following Endovascular Intervention for Peripheral Artery Disease Using Extreme Gradient Boosting (XGBoost) Model at the Preoperative Stage With Subgroup Analysis Based on Primary Procedure Type

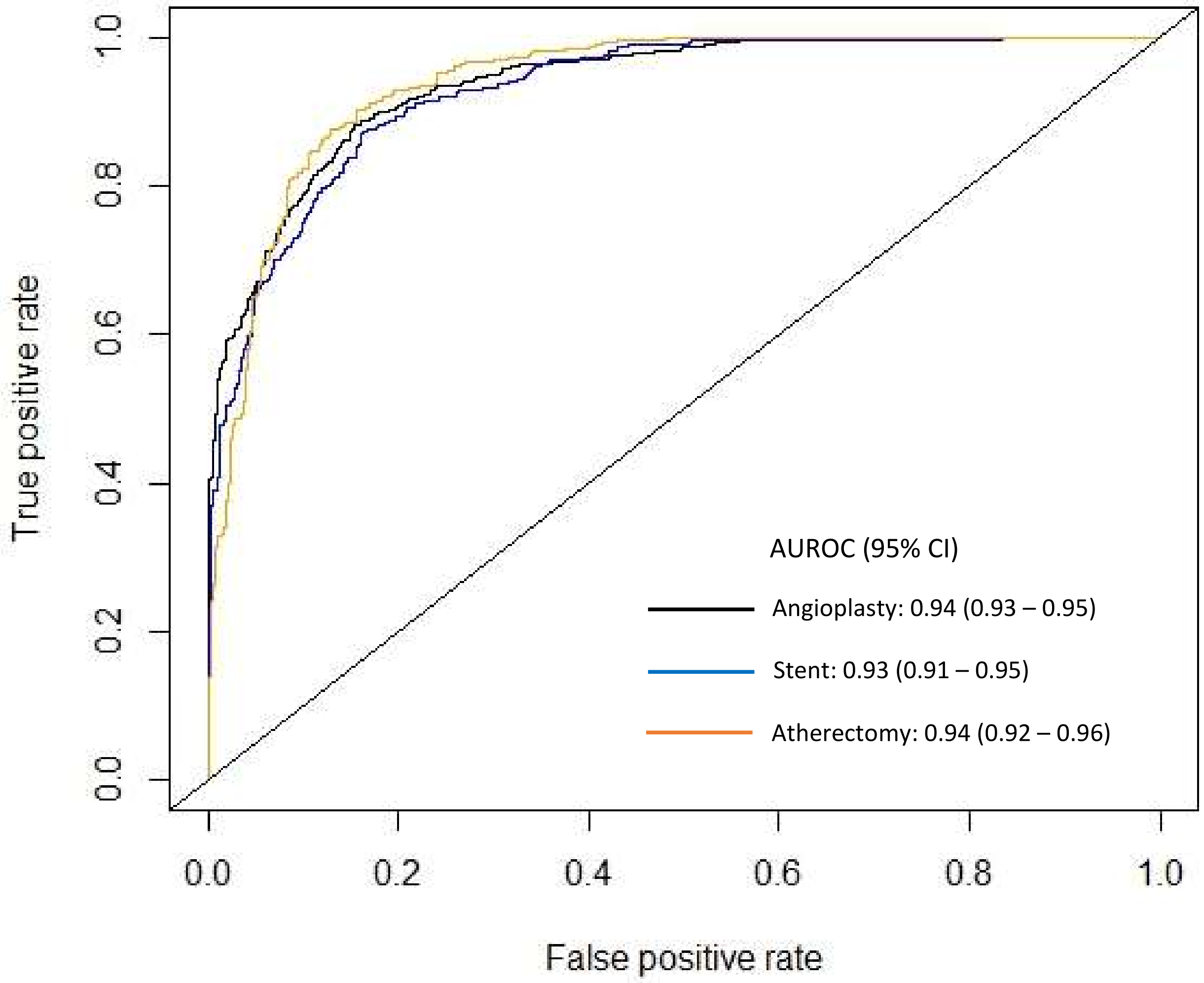

AUROC (area under the receiver operating characteristic curve), CI (confidence interval).

© 2024 Li B et al. *JAMA Network Open.*

eFigure 11. Receiver Operating Characteristic Curve for Predicting 1-Year Major Adverse Limb Event or Death Following Endovascular Intervention for Peripheral Artery Disease Using Extreme Gradient Boosting (XGBoost) Model at the Preoperative Stage With Subgroup Analysis Based on Location of Primary Artery Treated

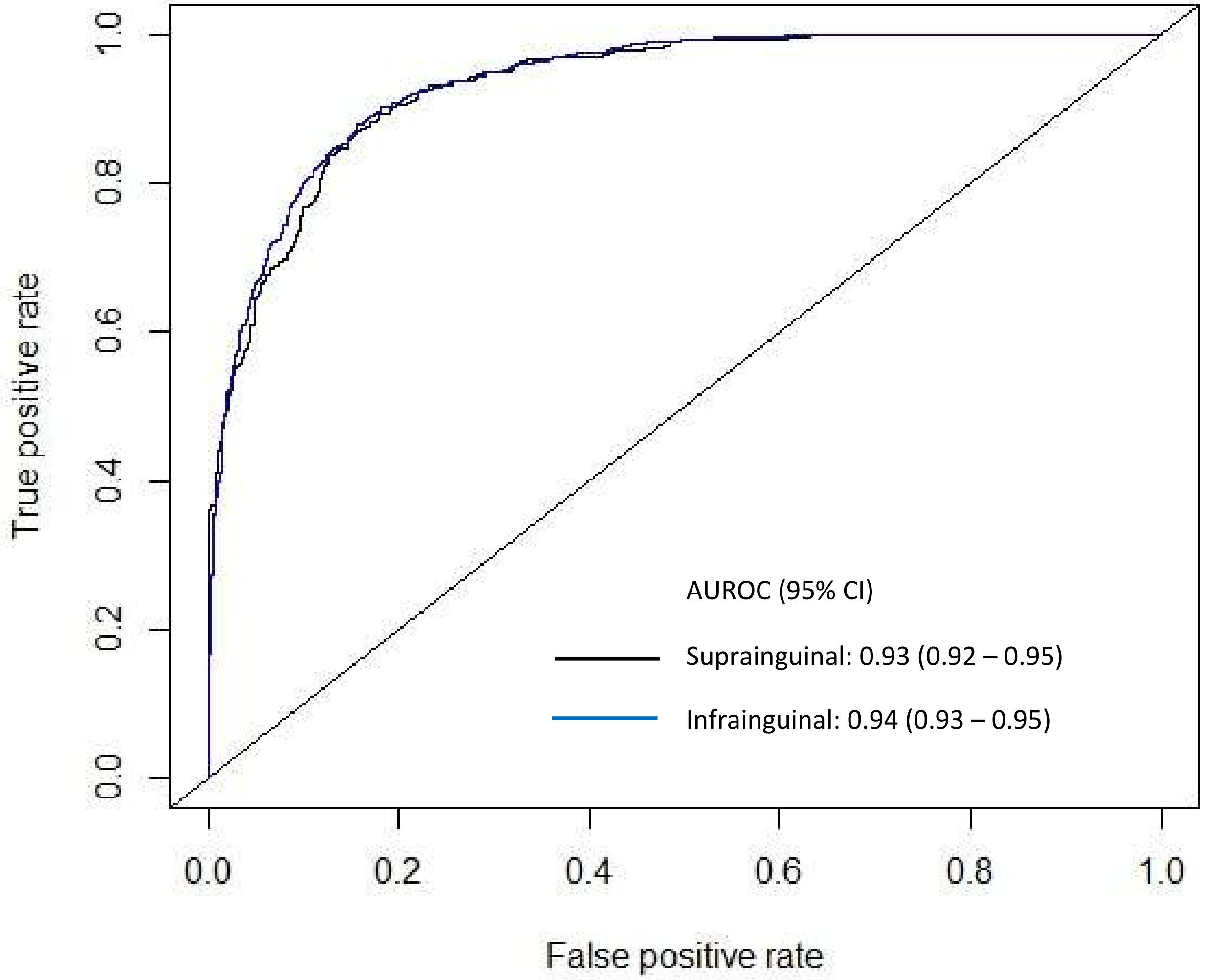

AUROC (area under the receiver operating characteristic curve), CI (confidence interval).

© 2024 Li B et al. *JAMA Network Open.*

eFigure 12. Receiver Operating Characteristic Curve for Predicting 1-Year Major Adverse Limb Event or Death Following Endovascular Intervention for Peripheral Artery Disease (PAD) Using Extreme Gradient Boosting (XGBoost) Model at the Preoperative Stage With Subgroup Analysis Based on Presence of Prior Open or Endovascular Intervention for PAD

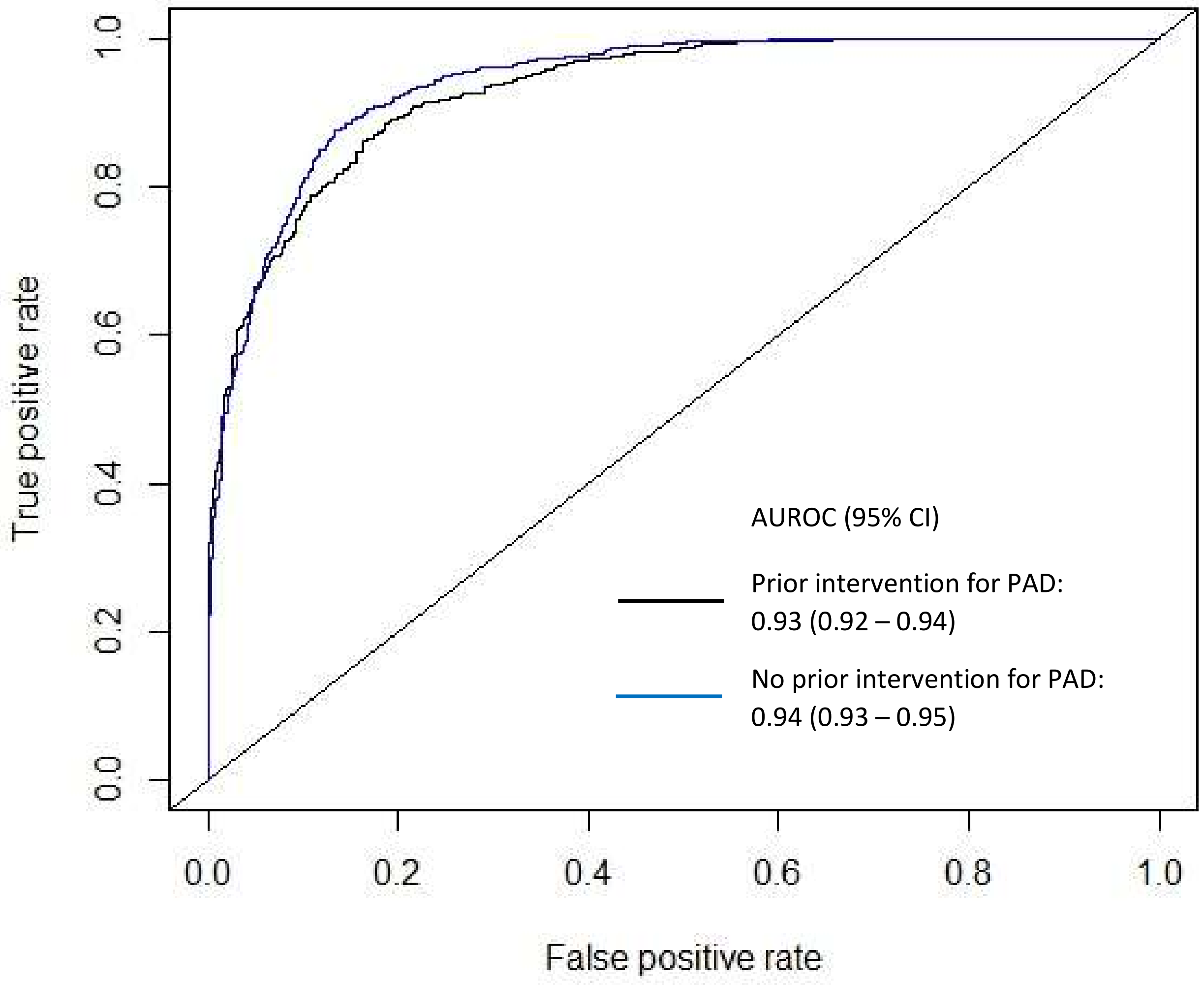

AUROC (area under the receiver operating characteristic curve), CI (confidence interval).

© 2024 Li B et al. *JAMA Network Open.*

eFigure 13. Receiver Operating Characteristic Curve for Predicting 1-Year Major Adverse Limb Event or Death Following Endovascular Intervention for Peripheral Artery Disease (PAD) Using Extreme Gradient Boosting (XGBoost) Model at the Preoperative Stage With Subgroup Analysis Based on Procedure Setting

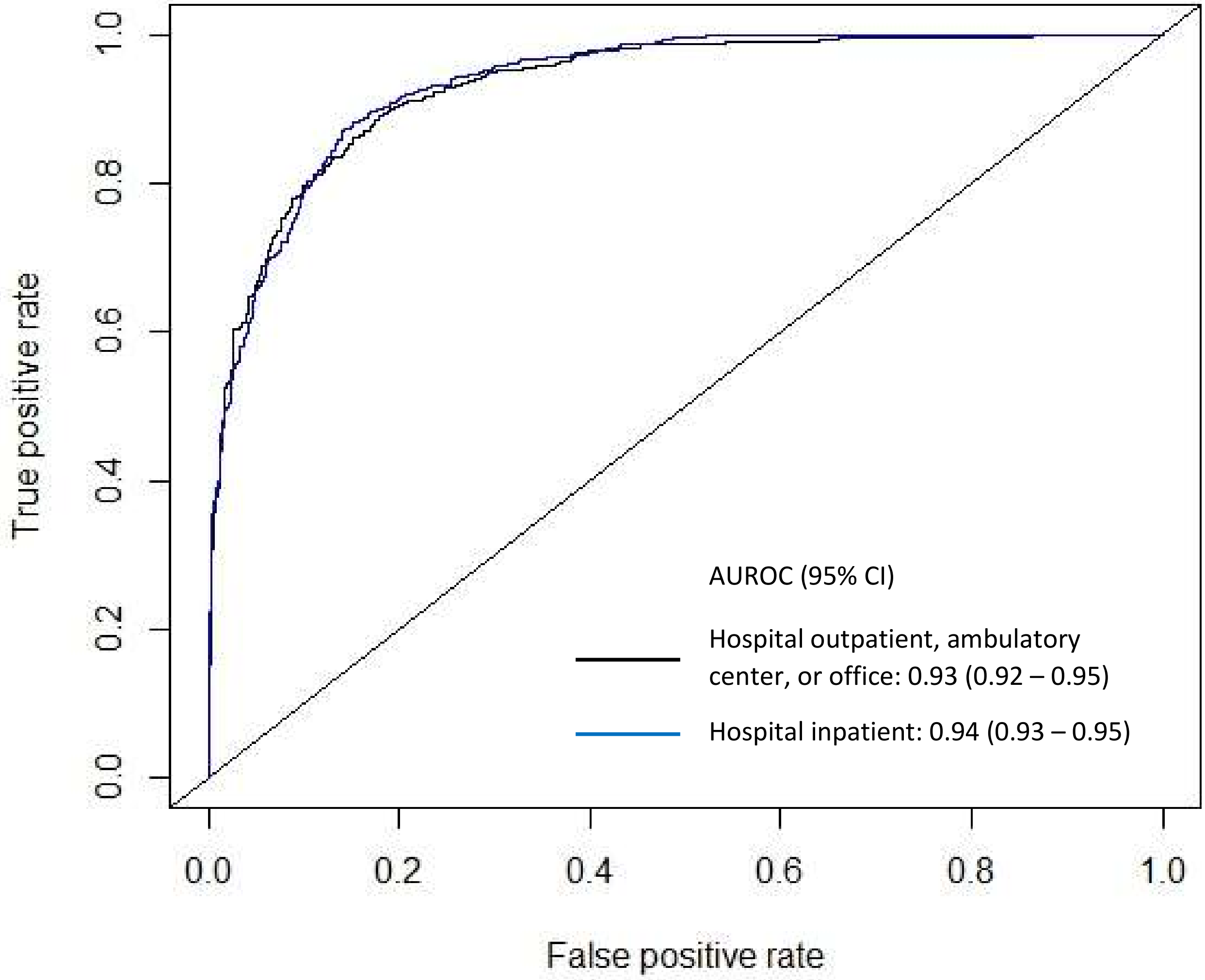

AUROC (area under the receiver operating characteristic curve), CI (confidence interval).

© 2024 Li B et al. *JAMA Network Open.*

eFigure 14. Receiver Operating Characteristic Curve for Predicting 1-Year Major Adverse Limb Event or Death Following Endovascular Intervention for Peripheral Artery Disease (PAD) Using Extreme Gradient Boosting (XGBoost) Model at the Preoperative Stage With Subgroup Analysis Based on Urgency

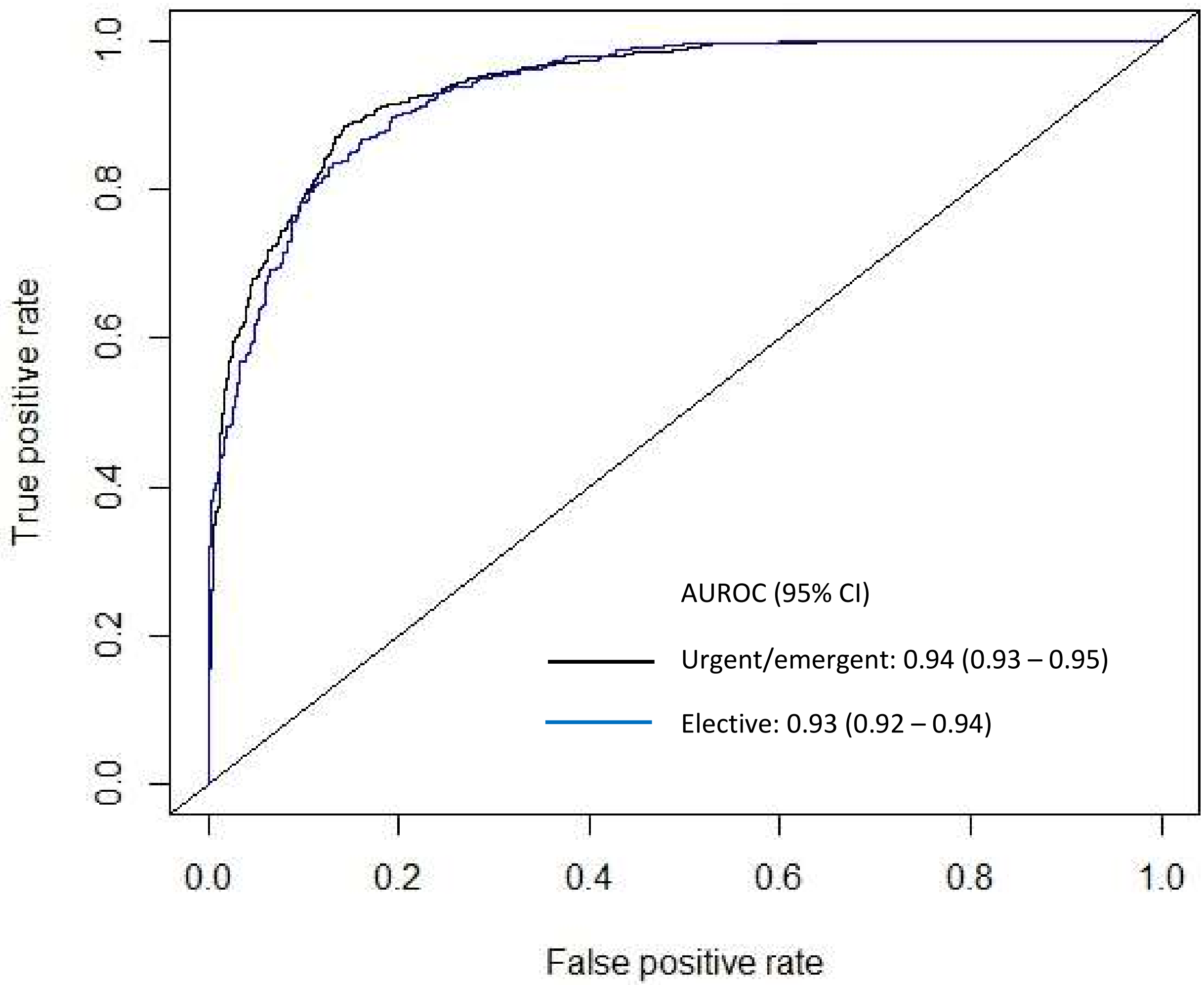

AUROC (area under the receiver operating characteristic curve), CI (confidence interval).

© 2024 Li B et al. *JAMA Network Open.*
