# Supplementary material for: Machine Learning to Predict Outcomes of Endovascular Intervention for Patients With PAD
Source: JAMA Netw Open. 2024 Mar 14;7(3):e242350. doi: 10.1001/jamanetworkopen.2024.2350 (PMC10940965; doi:10.1001/jamanetworkopen.2024.2350)
Supplement: Supplement 2. — Data Sharing Statement [file jamanetwopen-e242350-s002.pdf]

Data Sharing Statement

Li. Machine Learning to Predict Outcomes of Endovascular Intervention for Patients With PAD. *JAMA Netw Open*. Published March 14, 2024. doi:10.1001/jamanetworkopen.2024.2350

Data
Data available: Yes
Data types: Deidentified participant data
How to access data: The data used for this study comes from the Vascular Quality Initiative Database, which is maintained by the Society for Vascular Surgery Patient Safety Organization. Access and use of the data requires approval through an application process available at https://www.vqi.org/data-analysis/.
When available: With publication

Supporting Documents
Document types: Statistical/analytic code
How to access documents: The complete code used for model development and evaluation in this project is publicly available on GitHub: https://github.com/benli12345/PVI-ML-VQI.
When available: With publication

Additional Information
Who can access the data: Public
Types of analyses: Any purpose
Mechanisms of data availability: Public
